# COVID-19 and RA share an SPP1 myeloid pathway that drives PD-L1^+^ neutrophils and CD14^+^ monocytes

**DOI:** 10.1172/jci.insight.147413

**Published:** 2021-06-18

**Authors:** Lucy MacDonald, Stefano Alivernini, Barbara Tolusso, Aziza Elmesmari, Domenico Somma, Simone Perniola, Annamaria Paglionico, Luca Petricca, Silvia L. Bosello, Angelo Carfì, Michela Sali, Egidio Stigliano, Antonella Cingolani, Rita Murri, Vincenzo Arena, Massimo Fantoni, Massimo Antonelli, Francesco Landi, Francesco Franceschi, Maurizio Sanguinetti, Iain B. McInnes, Charles McSharry, Antonio Gasbarrini, Thomas D. Otto, Mariola Kurowska-Stolarska, Elisa Gremese

**Affiliations:** 1 Research into Inflammatory Arthritis Centre Versus Arthritis (RACE), University of Glasgow, United Kingdom.; 2 Division of Rheumatology, Fondazione Policlinico Universitario Agostino Gemelli IRCCS, Rome, Italy.; 3 Division of Rheumatology, Università Cattolica del Sacro Cuore, Rome, Italy.; 4 Institute of Internal Medicine and Geriatrics and; 5 Dipartimento di Scienze di Laboratorio e Infettivologiche, Fondazione Policlinico Universitario Agostino Gemelli IRCCS, Rome, Italy.; 6 Dipartimento di Scienze Biotecnologiche di Base, Cliniche Intensivologiche e Perioperatorie – Sezione di Microbiologia, Università Cattolica del Sacro Cuore, Rome, Italy.; 7 Department of Woman and Child Health and Public Health, Area of Pathology, and U.O.S.D. Coordinamento attività di Settorato, and; 8 Dipartimento di Scienze di Laboratorio e Infettivologiche, Fondazione Policlinico Universitario Agostino Gemelli IRCCS, Rome, Italy.; 9 Dipartimento di Sicurezza e Bioetica, Sez. Malattie Infettive, Università Cattolica del Sacro Cuore, Rome, Italy.; 10 Emergency Medicine, Fondazione Policlinico Universitario Agostino Gemelli IRCCS, Rome, Italy.; 11 Emergency Medicine, Università Cattolica del Sacro Cuore, Rome, Italy.; 12 Dipartimento di Scienze dell’Emergenza, Anestesiologiche e della Rianimazione, Fondazione, Policlinico Universitario Agostino Gemelli IRCCS, Rome, Italy.; 13 Dipartimento di Scienze dell’Emergenza, Anestesiologiche e della Rianimazione, Università Cattolica del Sacro Cuore, Rome, Italy.; 14 Institute of Infection, Immunity and Inflammation, University of Glasgow, United Kingdom.; 15 Dipartimento di Scienze Mediche e Chirurgiche, Fondazione Policlinico Universitario Agostino Gemelli IRCCS, Rome, Italy.; 16 Dipartimento di Medicina e Chirurgia Traslazionale, Università Cattolica del Sacro Cuore, Rome, Italy.

**Keywords:** Infectious disease, Inflammation, Autoimmune diseases, Macrophages

## Abstract

We explored the potential link between chronic inflammatory arthritis and COVID-19 pathogenic and resolving macrophage pathways and their role in COVID-19 pathogenesis. We found that bronchoalveolar lavage fluid (BALF) macrophage clusters FCN1^+^ and FCN1^+^SPP1^+^ predominant in severe COVID-19 were transcriptionally related to synovial tissue macrophage (STM) clusters CD48^hi^S100A12^+^ and CD48^+^SPP1^+^ that drive rheumatoid arthritis (RA) synovitis. BALF macrophage cluster FABP4^+^ predominant in healthy lung was transcriptionally related to STM cluster TREM2^+^ that governs resolution of synovitis in RA remission. Plasma concentrations of SPP1 and S100A12 (key products of macrophage clusters shared with active RA) were high in severe COVID-19 and predicted the need for Intensive Care Unit transfer, and they remained high in the post–COVID-19 stage. High plasma levels of SPP1 were unique to severe COVID-19 when compared with other causes of severe pneumonia, and IHC localized SPP1*^+^* macrophages in the alveoli of COVID-19 lung. Investigation into SPP1 mechanisms of action revealed that it drives proinflammatory activation of CD14*^+^* monocytes and development of PD-L1*^+^* neutrophils, both hallmarks of severe COVID-19. In summary, COVID-19 pneumonitis appears driven by similar pathogenic myeloid cell pathways as those in RA, and their mediators such as SPP1 might be an upstream activator of the aberrant innate response in severe COVID-19 and predictive of disease trajectory including post–COVID-19 pathology.

## Graphical abstract

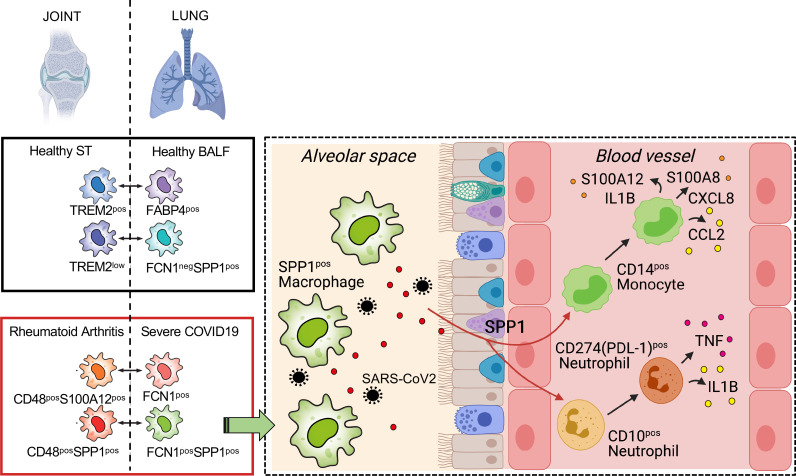

## Introduction

The severity of acute COVID-19 is attributable to genetics, immune dysregulation, abnormal blood clotting and tissue disruption, particularly implicating aberrant proinflammatory and antiviral innate immunity (1–14). Rheumatoid arthritis (RA) represents a proinflammatory cytokine–driven chronic articular condition often accompanied by interstitial lung disease and alveolitis (15). We (16) and others (17) have reported that SARS-CoV-2 infection is associated with emergence of polyarthritis, or flares of synovitis in RA patients in sustained disease remission, suggesting shared mechanisms of pathogenesis. RA immunopathogenesis and therapeutic targets (18) are well understood and might be informative for COVID-19 therapeutic strategies. In RA, articular inflammation and remission are driven by distinct synovial tissue macrophage clusters (19). Emerging data suggest that similar aberrant activation of myeloid cells in the blood (7, 20–23) and lung (24, 25) contribute to acute COVID-19 severity. However, there remains a knowledge gap on myeloid cell pathways that determine the severity and resolution of acute COVID-19 pneumonitis. In addition, the immune mechanisms of long-lasting clinical sequelae (26) described in convalescent COVID-19 patients are unresolved, hindering development of effective treatments and biomarkers of disease trajectory.

Single-cell profiling and fate-mapping indicate spatial and functional macrophage heterogeneity that maintains lung homeostasis (27–31). Healthy lung alveolar macrophages (AM) expressing *Fatty Acid Binding Protein 4* (*FABP4*) localize to the alveolar epithelial surface and recycle surfactants with type-2 alveolar epithelial cells to maintain compliance and efficient gas exchange (32, 33). This function is compromised in severe COVID-19 (34). The single-cell RNA sequencing (scRNAseq) analysis (24) of bronchoalveolar lavage fluid (BALF) from severe COVID-19 patients found abnormally low numbers of resident AM and an increase in 2 macrophage clusters that share expression of ficolin-1 (FCN1) and are distinguished by their relative expression of osteopontin (SPP1): FCN1*^+^* and FCN1*^+^*SPP1*^+^* clusters. Their precise roles in the pathogenesis of severe COVID-19 have yet to be established. We recently identified similar macrophage diversity in synovial tissues (ST) of healthy donors and patients with active or remission RA (19). We delineated in RA chronic synovitis that the CD48^hi^S100A12*^+^* and CD48*^+^*SPP1*^+^* macrophage clusters produced their distinctive pathogenic mediators (S100A12 and SPP1, respectively) in addition to hallmark proinflammatory cytokines IL-6, IL-8, IL-1β, and TNF-α, and activated ST stromal cells. Early evidence suggested that this serum IL-6, IL-8, IL-1β, and TNF-α cytokine signature also predicts the prognosis of patients with acute COVID-19 (6), and targeting the IL-6 pathway seems an effective therapeutic strategy in critically ill COVID-19 patients (35, 36). Resolution of synovitis in RA was associated with the functions of a distinct subpopulation of synovial macrophages (TREM2*^+^* and FOLR2*^+^* clusters) that produced abundant lipid resolvins instead of inflammatory mediators and induced a repair phenotype in ST stromal cells (19). Altogether, this raised the hypothesis that macrophage clusters in the lung, functionally equivalent to those in the synovium, may govern chronic inflammation or resolution of COVID-19 pneumonitis, and that the hallmark cytokine signature related to these clusters (e.g., SPP1) might be a useful biomarker of prognosis and a therapeutic target during the unpredictable trajectory of COVID-19.

## Results

### COVID-19 BALF FCN1^+^ and FCN1^+^SPP1^+^ macrophage clusters are transcriptionally similar to CD48^hi^S100A12^+^ and CD48^+^SPP1^+^ clusters that drive RA synovitis.

**Figure 1:**
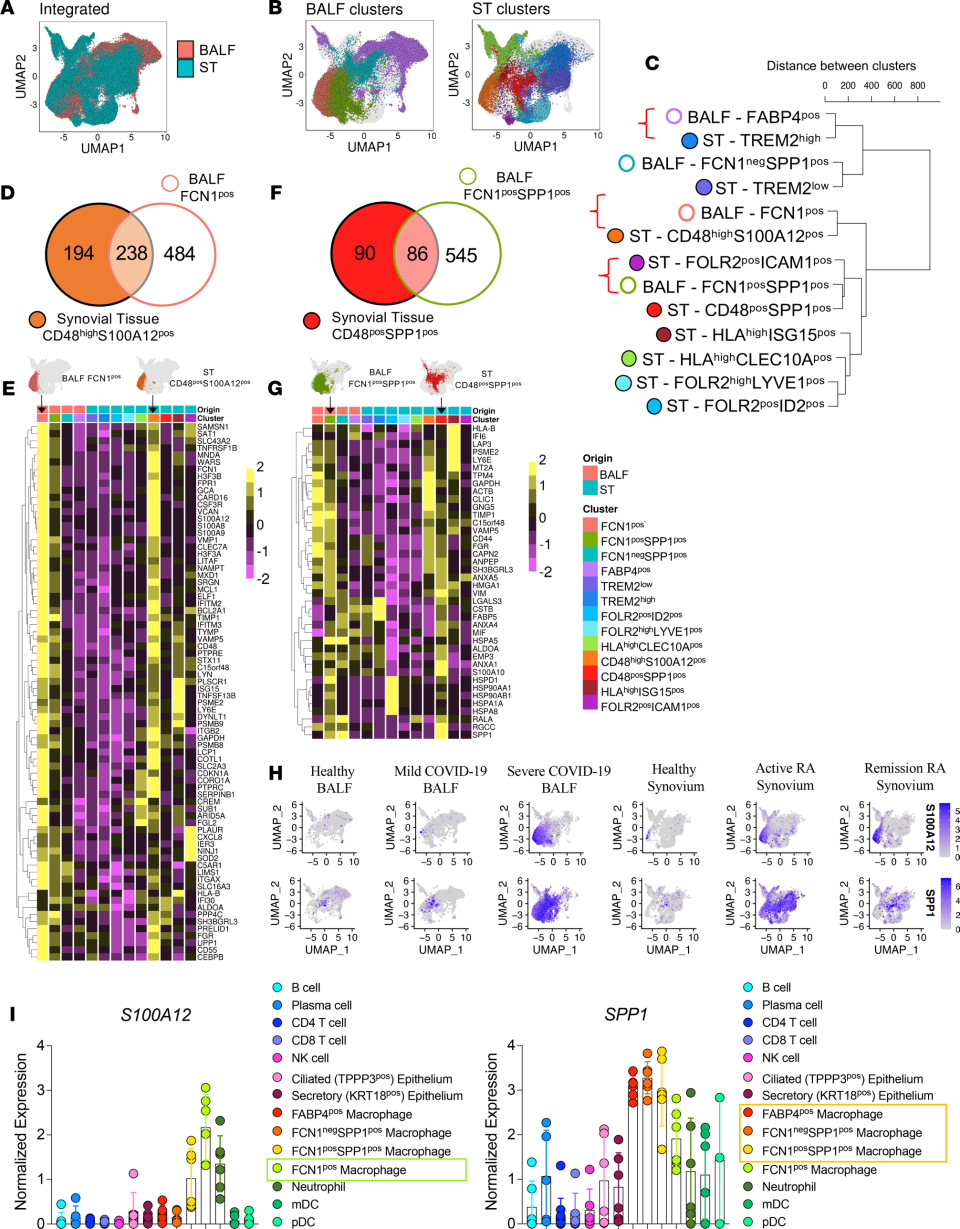
COVID-19 BALF FCN1^+^ and FCN1^+^SPP1^+^ macrophage clusters are transcriptionally related to rheumatoid arthritis synovial CD48^hi^S100A12^+^ and CD48^+^SPP1^+^ macrophage clusters. (**A** and **B**) UMAP of an equal number of synovial tissue and BALF macrophages (32,000 from each data set), colored according to their ST and BALF cluster identification. (**C**) Dendrogram of hierarchical clustering analysis of integrated pseudobulk gene expression (average expression in each cluster) of ST and BALF clusters. (**D**) Venn diagram illustrating the numbers of unique and shared marker genes of ST CD48^hi^S100A12^+^ and BALF FCN1^+^ clusters. *P* < 0.05 after Bonferroni correction for multiple comparisons. (**E**) Heatmap illustrating scaled, pseudo-bulk gene expression of shared upregulated marker genes (highlighted in **D**) by BALF and ST clusters. (**F**) Venn diagram illustrating numbers of unique and shared marker genes of ST CD48^+^SPP1^+^ and BALF FCN1^+^SPP1^+^ clusters generated as in **E**. (**G**) Heatmap illustrating scaled, pseudobulk gene expression of shared upregulated marker genes (highlighted in **F**) by ST and BALF clusters. (**H**) Split UMAP plots comparing *S100A12* and *SPP1* expression in BALF and ST macrophage clusters across different conditions. Intensity of purple indicates expression level. HC, healthy control. (**I**) Dot plots illustrating normalized (mean ± SEM) expression values of *S100A12* and *SPP1* per cell across all immune and epithelial cell clusters in severe COVID-19 BALF (*n =* 6). Framed populations showed the highest expression of these markers.

In COVID-19 BALF, Liao et al. (24) identified 4 major clusters of macrophages characterized by a combination of expression of SPP1, FCN1, and FABP4. Importantly, expansion of the FCN1*^+^* and FCN1^+^SPP1^+^ clusters was indicative of COVID-19 severity (Supplemental Figure 1, A and B; supplemental material available online with this article; https://doi.org/10.1172/jci.insight.147413DS1). Among the 9 phenotypically distinct clusters of ST macrophages (STMs) that differed in distribution between healthy donors and patients with RA, the CD48^hi^S100A12^+^ and CD48^+^SPP1^+^ clusters were expanded in RA patients with active disease (Supplemental Figure 1, C and D) (19). To test the relationship between COVID-19 BALF and RA STM clusters, we integrated the macrophage scRNAseq data sets from COVID-19 BALF (*GSE145926*; Supplemental Figure 1, A and B) (24) and our RA ST (*E-MTAB-8322;* Supplemental Figure 1, C and D) (19) using Seurat V3 anchor integration strategy (37). The dimensional reduction of integrated macrophage data sets illustrated by Uniform Manifold Approximation and Projection (UMAP) visualize overlapping and tissue-specific macrophage clusters (Figure 1, A and B). To investigate the relationship between these macrophage clusters from synovitis and pneumonitis, hierarchical clustering (Figure 1C) was performed on the matrix of the average expression of each ST and BALF cluster of all 8902 genes that were common to both data sets. The hierarchical clustering dendrogram illustrating the relationship between the clusters by branch point (split) and branch length (distance) revealed that the macrophage clusters were separated predominantly by their precursor origin and function — i.e., embryonic-origin (homeostasis) or monocyte-derived (inflammation) rather than source tissue. The first branch point separated 4 tissue-resident macrophage clusters (healthy lung FABP4^+^ and FABP4^lo^FCN^–^SPP1^+^, and synovial lining TREM2^hi^ and TREM2^lo^) from the other BALF and STM clusters that were likely of monocytic origin (19, 24, 25). The split-points and distances in the second branch of hierarchical clustering indicate that the proinflammatory clusters (i.e., FCN1^+^ and FCN1^+^SPP1^+^) that dominate in severe COVID-19 share transcriptomic profiles with proinflammatory clusters (i.e., CD48^hi^S100A12^+^ and CD48^+^SPP1^+^, respectively) in active RA. Pearson correlation analysis between BALF FCN1^+^ and ST CD48^hi^S100A12^+^, and between BALF FCN1^+^SPP1^+^ and ST CD48^+^SPP1^+^, confirmed resemblance (*r* = 0.56, *P =* 2.2 × 10^–16^ and *r* = 0.65, *P =* 2.2 × 10^–16^) of respective BALF/ST pairs. Independent differential gene expression analysis identified striking similarities in the pathogenic pathways of COVID-19 pneumonitis FCN1^+^ and active RA synovitis CD48^hi^S100A12^+^ clusters. They share 238 top marker genes (Figure 1D) that include upregulation of inflammatory and prothrombotic pathways. These consisted of IFN pathway (e.g.*, IFITM2*, *IFITM3*, and *ISG15*), inflammation-triggering alarmins (*S100A8/9/12A*), B cell activation factors (e.g., BAFF-*TNFSF15*B), promotors of IL-1β and TNF production (e.g., *CARD16* and *LITAF*), prothrombotic factors (e.g., *FGL2*), and integrins mediating cell migration and adhesion (e.g., *ITGB2* and *ITGX*). They also share receptor expression profiles — e.g., TNFR2 (*TNFRSF1B*), G-CSFR (*CSF3R*), and the complement receptor (*C5aR*; ref. 38) — that may render them increasingly susceptible to proinflammatory mediators to further escalate inflammation (Figure 1E). The hierarchical analysis and comparison of cluster markers also indicate a shared transcriptional profile of COVID-19 FCN1^+^SPP1^+^ and synovitis CD48^+^SPP1^+^ clusters consisting of 86 common marker genes including *SPP1*, which have broad proinflammatory and fibrotic functions (Figure 1, F and G) (39). These transcriptomic profiles suggest that BALF FCN^+^ and FCN^+^SPP1^+^ macrophage clusters predominant in severe COVID-19 shared pathogenic molecular pathways, including the expression of their signature mediators *S100A12* and *SPP1* (Figure 1H) with ST CD48^hi^S100A12^+^ and CD48^+^SPP1^+^ clusters predominant in active RA. Analysis of *SPP1* and *S100A12* expression in other cell types present in lung tissue (Figure 1I and Supplemental Figure 2) demonstrated that BALF macrophage populations are the main cells expressing *SPP1* and *S100A12* in severe COVID-19, and due to their high numbers in BALF of severe COVID-19(Supplemental Figure 1), FCN^+^ and FCN^+^SPP1^+^ macrophage clusters are likely the main contributors to the S100A12 and SPP1 pool in the COVID lung.

### Healthy lung alveolar FABP4^+^ macrophages share a homeostatic regulatory transcriptomic profile, including the TAM pathway, with ST lining layer TREM2^hi^ macrophages.

**Figure 2:**
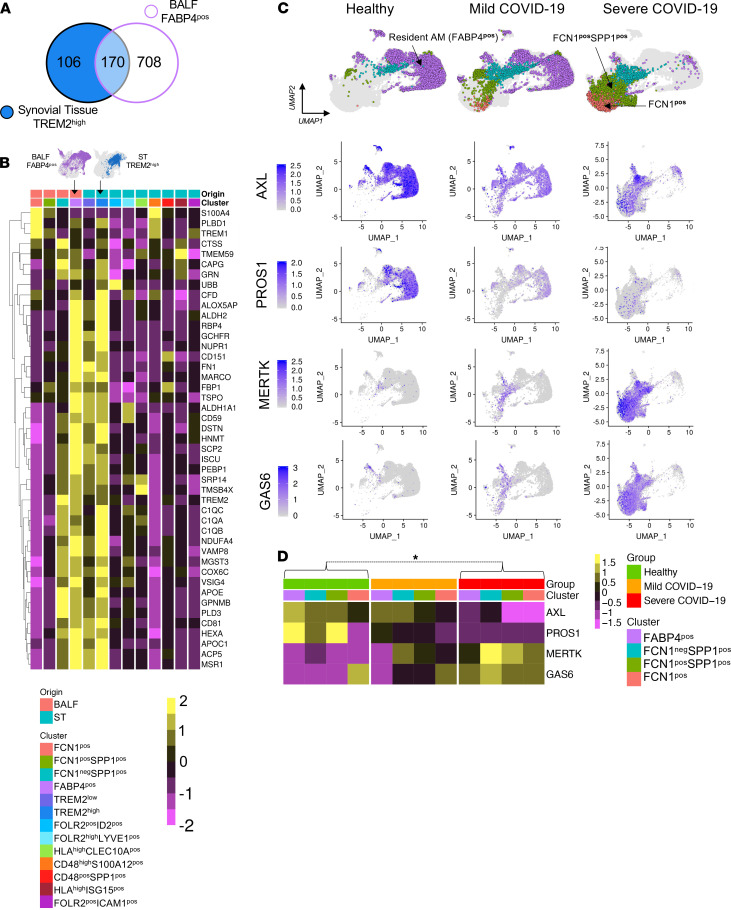
COVID-19 BALF FABP4^+^ and RA synovial TREM2^+^ macrophages share transcriptomic profiles and regulatory TAM receptor pathways. (**A**) Venn diagram illustrating numbers of unique and shared marker genes of ST TREM2^hi^ and BALF FABP4^+^ macrophage clusters as described in Figure 1. Marker genes were identified prior to integration of data sets (19, 24) and were calculated using MAST, setting a minimum percentage of cells in clusters expressing each marker to 40%. Genes considered differentially expressed at *P <* 0.05 after Bonferroni correction. (**B**) Heatmap illustrating scaled, pseudobulk expression of shared upregulated marker genes from ST and BALF clusters indicated in **A**. (**C**) Split UMAP plots comparing BALF macrophage clusters in health, and in mild and severe COVID-19, illustrating changes in expression of the TAM receptors *AXL* and *MerTK*, with their respective preferred ligands *GAS6* and *PROS1*. Intensity of purple indicates expression level. (**D**) Heatmap illustrating scaled, pseudobulk expression of TAM receptors and associated ligands by each BALF cluster, across patient groups. TAM receptors and their ligands were significantly differentially expressed in severe COVID-19 versus healthy tissues (*P* ≤ 0.005), with Bonferroni correction for multiple comparison, as confirmed by MAST.

The innate immune pathways that restrain inflammation in acute COVID-19 are yet to be characterized. Recently uncovered innate mechanisms reinstating ST homeostasis in RA (19) might shed new light on potential mechanisms of resolution for COVID-19. The ST of RA patients in sustained disease remission is dominated by macrophage clusters with protective and inflammation-resolving properties (19). Of these resolving clusters, ST TREM2^hi^ clustered tightly with the FABP4^+^ AM cluster in unsupervised hierarchical analysis (Figure 1C). Pearson correlation of 986 TREM2^+^ and FABP4^+^ unique and common cluster markers (*r* = 0.83 *P =* 2.2 × 10^–16^) confirmed the similarities. Differential expression analysis of their profiles identified that BALF FABP4^+^ and ST TREM2^hi^ clusters share 170 markers indicating analogous homeostatic functions (Figure 2, A and B). These include the complement pathways (e.g., *C1q)* that facilitates uptake of apoptotic bodies, high expression of genes of retinoic acid production (e.g.*,* *ALDH1A1* and *RBP4*) that drives Treg differentiation (40), and the B7-related coinhibitory molecule *VSIG4* that inhibits T effector cells (41); this suggests a primary role of this cluster in governing lung immunity (Figure 2B). While the functional contribution of FABP4^+^ macrophages to the resolution of SARS-CoV-2 infection is yet to be established, we have shown previously that their counterpart TREM2^+^ STM clusters produced inflammation-resolving lipid mediators and induced a repair phenotype in tissue stromal cells that maintain disease remission. These homeostatic responses were driven by MerTK, a member of the immunosuppressive tyrosine kinase receptor TAM family (TYRO, AXL, and MerTK; ref. 19). TAM receptors and their ligands GAS6 or the PROS1 gene product Protein S (PROS1) form a homeostatic brake on inflammation and autoimmunity (42–44). In addition, Protein S is an essential inhibitor of blood coagulation preventing thrombosis (45). Lung-resident FABP4 macrophages uniquely express AXL rather than MerTK. Their AXL is constitutively ligated to GAS6 (46, 47) and is key in preventing exacerbated inflammation — e.g., during influenza virus infection (46, 47). Analysis of the GSE145926 data set (24) identified profoundly altered macrophage expression of TAM receptors and their ligands in the lung (Figure 2, C and D) that might explain the inadequate regulation of the tissue hyperinflammatory and thrombotic responses in severe COVID-19. The TAM receptors and ligand pathway was mostly expressed by lung myeloid cells, with a contribution from ciliated epithelium to the lung Protein S pool (Supplemental Figures 2 and 3). AM from healthy lungs showed high expression levels of *AXL* and *PROS1*, and these were markedly reduced in patients with severe COVID-19 (Figure 2, C and D). *GAS6* and *MerTK* were not expressed by resident AMs; instead, they were increasingly expressed by infiltrating FCN^+^ and FCN^+^SPP1^+^ macrophage clusters, suggesting an inflammation-triggered attempt to counterbalance pathogenic responses. However, the reduced *PROS1*, which is the preferred activating ligand for MerTK (44, 48), and the fewer homeostatic resident AMs in severe COVID-19 might enable unrestricted proinflammatory cytokine production by locally differentiated MerTK-expressing FCN^+^ and FCN^+^SPP1^+^ macrophages.

### High plasma SPP1 is associated with a severe disease trajectory of COVID-19.

**Figure 3:**
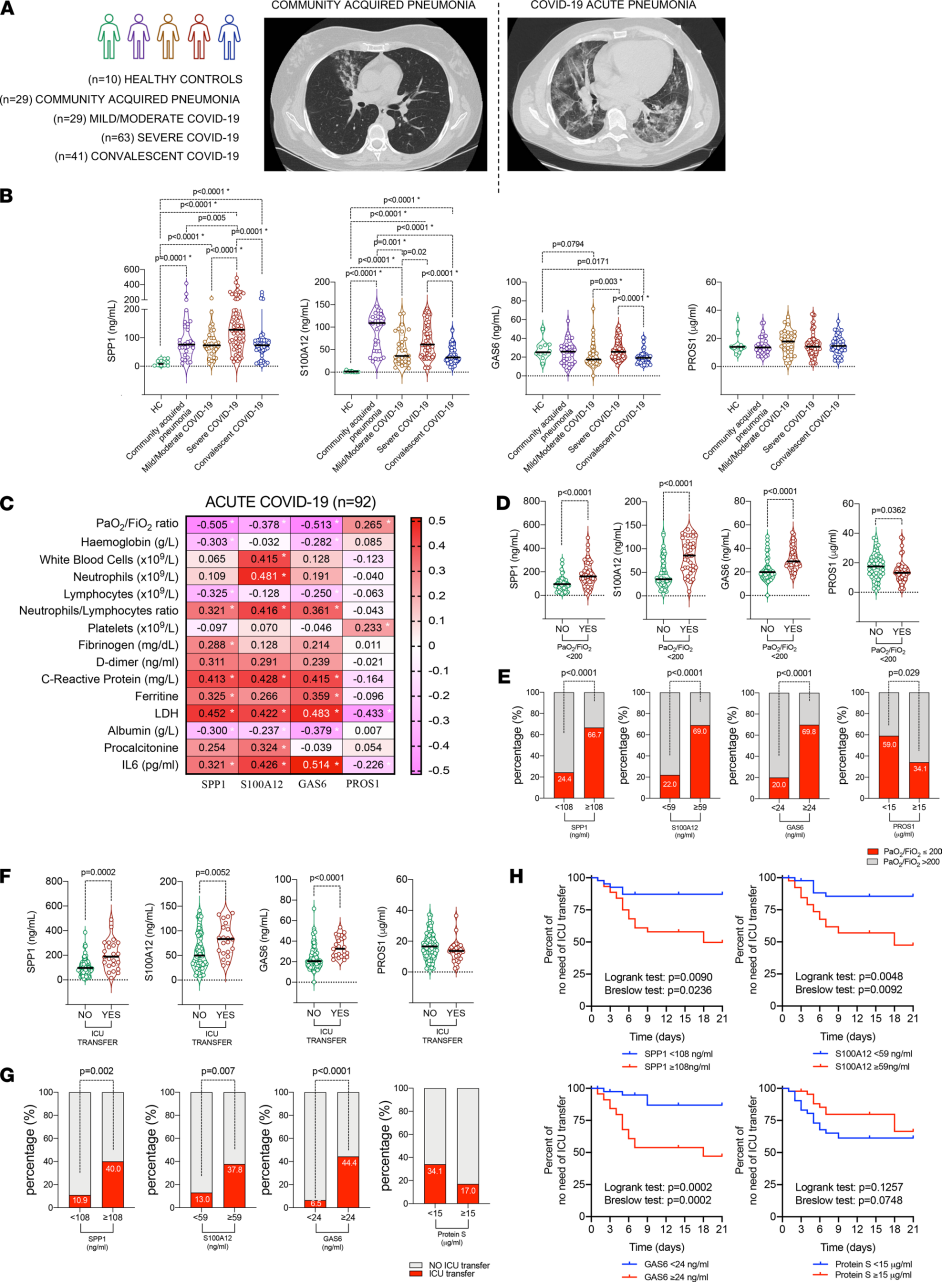
High levels of SPP1 and S100A12 are associated with a severe COVID-19 disease trajectory. (**A**) Patients and healthy donors, shown as the following: *n =* 121 patients with acute pneumonia (*n =* 29 community acquired SARS-CoV-2^–^ pneumonia, *n =* 29 mild/moderate COVID-19, *n =* 63 severe COVID-19), convalescent COVID-19 (*n =* 41), and healthy controls (*n =* 10). Representative images of lung CT scans. (**B**) Plasma levels of SPP1, S100A12, GAS6, and PROS1 in groups as in **A**. (**C**) Spearman’s rank correlations between SPP1, S100A12, GAS6, and PROS1 plasma levels in patients with acute COVID-19 pneumonia (*n =* 92) with demographic and clinical parameters. Each box displays the *r* value, and an asterisk indicates statistical significance of *P <* 0.05. (**D**) Plasma levels of SPP1, S100A12, GAS6, and PROS1 in patients with acute COVID-19 pneumonia (*n =* 92) stratified based on lung functions measured by PaO_2_/FiO_2_ at the time of hospital admission. Severe respiratory failure was defined by PaO_2_/FiO_2_ ≤ 200. (**E**) Percentage of acute COVID-19 pneumonia patients (*n =* 92) with PaO_2_/FiO_2_ ≤ 200 based on high plasma levels of SPP1 (≥108 ng/mL), S100A12 (≥59 ng/mL), GAS6 (≥24 ng/mL), and PROS1 (≥15 μg/mL). (**F**) COVID-19 patient plasma levels of SPP1, S100A12, GAS6, and PROS1 at the time of hospital admission (*n =* 92) stratified based on a patient’s subsequent need to be transferred to ICU. (**G**) Percentage of patients with acute COVID-19 pneumonia (*n =* 92) transferred to ICU during the hospitalization based on having high levels of SPP1 (≥108 ng/mL), S100A12 (≥59 ng/mL), GAS6 (≥24 ng/mL), and PROS1 (≥15 μg/mL) at the time of hospital admission. (**B**, **D**, and **F**) Data are presented as violin plots with median and interquartile range. Asterisk indicates 1-way ANOVA (Kruskal-Wallis test) with Dunn’s correction for multiple comparisons if more than 2 groups were compared (**B**), or 2-sided Mann-Whitney *U* was used when 2 groups were compared (**B** and **D**–**G**). (**H**) Kaplan-Meier analysis of the rate of transfer of COVID-19 patients to ICU based on their cut-off values for SPP1, S100A12, GAS6, and PROS1 at the time of hospital admission.

To validate the computational scRNAseq findings of shared pathogenic macrophage clusters in COVID-19 and RA, we quantified plasma concentrations of their key shared functional mediators SPP1 (osteopontin) and S100A12 (calgranulin C) in a cross-sectional comparison of additional patient groups. These consisted of (a) hospitalized acute COVID-19 patients (*n =* 92), (b) COVID-19 patients in their post–COVID-19 phase (convalescence) at outpatient clinics (*n =* 41), (c) hospitalized patients with non–SARS-CoV-2 community–acquired severe acute pneumonia (*n =* 29), and (d) healthy donors (*n =* 10; Figure 3A). Demographic and clinical information, and laboratory biomarkers are presented in Supplemental Table 1. Blood samples for groups (a) and (c) were taken on hospital admission or shortly afterward and before antiinflammatory treatment (see Methods).

Patients with acute COVID-19 had significantly higher plasma levels of proinflammatory SPP1 and S100A12 than healthy donors and patients at the post–COVID-19 stage (Supplemental Figure 4A). Based on clinical criteria (see Methods) and before antiviral/antiinflammatory treatment administration, the acute COVID-19 patients were categorized into those who subsequently developed either mild/moderate or severe disease. We found that the S100A12 plasma levels were higher in COVID-19 patients categorized as severe compared with mild/moderate, and compared with those in convalescence, and with healthy donors, but comparable with the levels in patients with community acquired pneumonia (Figure 3B). Interestingly, levels of SPP1 were uniquely higher in those with severe COVID-19 compared with any other groups, including severe pneumonia induced by other pathogens (Figure 3B).

We next investigated the relationship between plasma levels of SPP1 and S100A12, with systemic inflammation measured by blood cytometry and biochemistry, and lung function measured by PaO_2_/FiO_2_ tested around the time of blood sampling and before administration of antiinflammatory treatment (Supplemental Table 1). Plasma concentrations of SPP1 and S100A12 correlated negatively with PaO_2_/FiO_2_ and positively with inflammatory biomarkers — e.g., IL-6, CRP, and LDH (Figure 3C and Supplemental Table 1). Moreover, SPP1 and S100A12 each strongly correlated with the neutrophil/lymphocyte ratio, which itself is a prognostic biomarker for COVID-19 severity (Figure 3C) (49). Stratification of patients according to levels of PaO_2_/FiO_2_ reflecting those with or without severe respiratory distress (PaO_2_/FiO_2_ ≤ 200 and PaO_2_/FiO_2_ > 200, respectively) clearly showed that the former had significantly higher levels of SPP1 and S100A12 (Figure 3D). A refinement of this observation suggested that COVID-19 patients with plasma levels of SPP1 ≥ 108 ng/mL and S100A12 ≥ 59 ng/mL (cut-off values based on their medians in all COVID-19 patients) were predictive of those more likely to have severe respiratory distress (PaO_2_/FiO_2_ ≤ 200) compared with those with lower SPP1 and S100A12 levels (Figure 3E and Supplemental Figure 4B). Of prognostic importance for COVID-19 patients, higher levels of SPP1 and S100A12 at the time of hospital admission and before antiinflammatory treatment were predictive of urgency for subsequent Intensive Care Unit (ICU) transfer (Figure 3, F and G). The time to ICU transfer was significantly more rapid for patients with plasma SPP1 ≥ 108 ng/mL and S100A12 ≥ 59 ng/mL (Figure 3H). These data suggest that a plasma biomarker signature associated with pathogenic macrophage clusters in the lung and shared with RA synovitis (i.e., SPP1 and S100A12) might be useful for predicting the trajectory of disease severity and indicative of mechanism of pathogenesis of severe COVID-19. Analysis of the SPP1 (CD44/integrins) and S100A12 (TLR4/CD36) receptor distribution in the lungs of COVID-19 patients demonstrated that many cell types expressed receptors for SPP1, including macrophages, neutrophils, T cells, and epithelial cells, whereas predominantly macrophages expressed receptors for S100A12 (Supplemental Figure 5). This suggested that the lungs acted as a receptive environment for the actions of proinflammatory SPP1 and S100A12.

We next investigated the plasma concentrations of COVID-19/RA shared regulatory TAM receptor pathway ligands GAS6 and PROS1 in the patient groups described above. They showed a distinct plasma signature compared with proinflammatory SPP1 and S100A12. At the time of acute pneumonia, plasma levels of GAS6, the preferable ligand for the AXL receptor, did not differ between acute severe COVID-19 and healthy donors. However, GAS6 levels were lower in patients with milder acute COVID-19 and in post–COVID-19 subjects in whom acute inflammation had resolved, compared with healthy donors and patients with severe COVID-19 (Figure 3B). Consistent with lower levels of GAS6 in patients with mild/moderate disease, GAS6 levels in COVID-19 patients correlated negatively with PaO_2_/FiO_2_ ratio (Figure 3C). COVID-19 patients with PaO_2_/FiO_2_ > 200 had lower plasma GAS6 levels than those with PaO_2_/FiO_2_ ≤ 200 (Figure 3D), and optimum cut-off plasma levels of GAS6 < 24 ng/mL detected those more likely to maintain good lung function (PaO_2_/FiO_2_ > 200) (Figure 3E). Prognostically, low levels of GAS6 at the time of hospital admission and/or before antiinflammatory treatment were predictive of low risk for ICU transfer (Figure 3F); for example, 6.5% of COVID-19 patients with GAS6 < 24 ng/mL required transfer to ICU compared with 44.4% of those with GAS6 ≥ 24 ng/mL (Figure 3, G and H). These data suggest that lower GAS6 levels were predictive of better disease outcomes. GAS6 can be produced by many immune cells (45, 50), including lung-infiltrating inflammatory FCN1^+^ and FCN^+^SPP1^+^ macrophages and DCs (Figure 2C and Supplemental Figure 2). Thus, low levels of plasma GAS6 in patients with milder disease may reflect a lower degree of lung infiltration by inflammatory macrophages.

Plasma level of PROS1, a marker of healthy lung *FABP4^+^* macrophages and the preferable ligand for MerTK, did not differ between any of the COVID-19 patient categories or healthy donors (Figure 3B). However, the levels of PROS1 in COVID-19 patients with mild/moderate disease showed a wider spread of the values as illustrated by interquartile range compared with other groups, suggesting potential association between PROS1 and individual composites of the classification criteria. In contrast to SPP1, A100A12, and GAS6, PROS1 levels correlated positively with PaO_2_/FiO_2_ and negatively with IL-6 (Figure 3C). COVID-19 patients with PaO_2_/FiO_2_ > 200 had higher plasma levels of PROS1 than those with PaO_2_/FiO_2_ ≤ 200 (Figure 3D). An optimum plasma PROS1 cut-off value was calculated at 15 μg/mL, above which 34.1% of COVID-19 patients had severe respiratory distress as compared with 59% of those with PROS1 < 15 μg/mL (Figure 3E). This observation is consistent with the single-cell transcriptomic data (Figure 2C) showing that the expression of inflammation resolving, antithrombotic PROS1 and antithrombotic PROS1 in resident alveolar FABP4^+^ macrophages decreases with increasing disease severity.

Next, we investigated whether concomitant pharmacological drug treatment, demographic factors (age and sex), and COVID-19 comorbidities (6) contributed to the plasma levels of SPP1, S100A12, GAS6, and PROS1. None of the pharmacological treatments affected the plasma levels of SPP1, S100A12, GAS6, and PROS1 in moderate and severe COVID-19 pneumonia (Supplemental Figure 6). S100A12 and PROS1 were unrelated to age in COVID-19 and in SARS-CoV-2^–^ pneumonia (Supplemental Figure 4, C–E). SPP1 was unaffected by age in SARS-CoV-2^–^ pneumonia but was higher in COVID-19 patients > 70 years old (Supplemental Figure 4, C–E), suggesting that SPP1 levels are related more to severity of pneumonia than age (Figure 3). GAS6 was higher in COVID-19 and non–SARS-CoV-2 pneumonia patients > 70 years old and higher in COVID-19 patients with arterial hypertension, diabetes mellitus, and ischemic cardiopathy (Supplemental Figure 7); thus, age and comorbidities may confound interpretation of increased GAS6 in COVID-19.

### Increased SPP1 levels persist after COVID-19.

**Figure 4:**
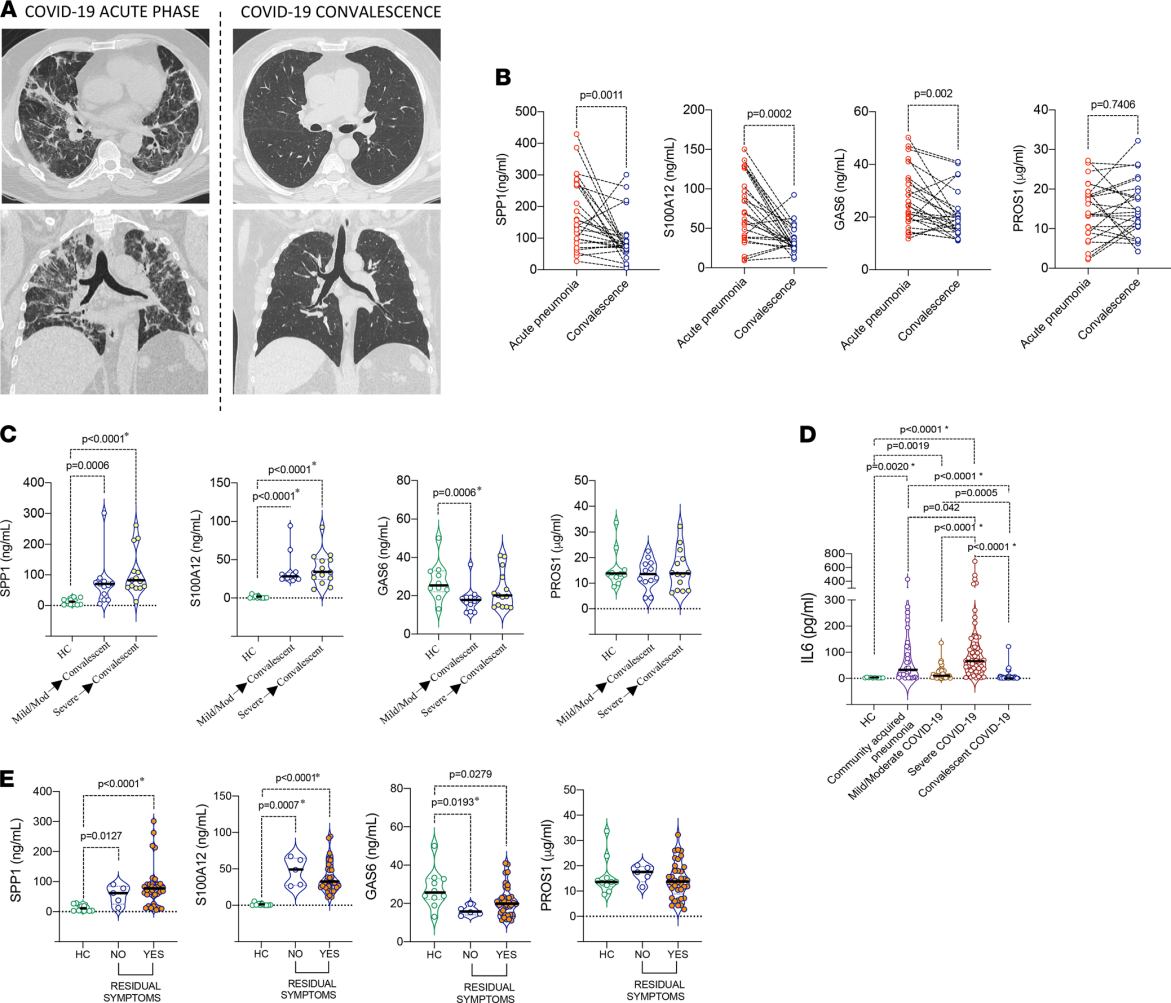
Increased SPP1 and S100A12 levels persist in post–COVID-19 phase. (**A**) Representative images of lung CT scans (transversal and sagittal view) of a COVID-19 patient taken during acute pneumonia and during convalescence (68.60 ± 4.36 days after hospital discharge). (**B**) Plasma levels of SPP1, S100A12, GAS6, and PROS1 in paired plasma samples from COVID-19 patients at the time of acute pneumonia and at the convalescent phase (*n =* 26). (**C**) Plasma levels of SPP1, S100A12, GAS6, and PROS1 in convalescent COVID-19 patients (*n =* 41) stratified based on the severity of prior acute pneumonia and compared with the levels of healthy donors (*n =* 10). (**D**) Plasma levels of IL-6 in acute pneumonias and post–COVID-19. (**E**) SPP1, S100A12, GAS6, and PROS1 in convalescent COVID-19 patients (*n =* 41) stratified based on suffering (*n =* 36) or not (*n =* 5) at least 1 of the symptoms (fatigue, musculoskeletal, or respiratory symptoms). (**B**) Data are presented as before-and-after plot. Wilcoxon test on paired samples was used, and exact *P* values are provided on the graphs. (**C**–**E**) Data are presented as violin plots with median and interquartile range. Asterisks indicate 1-way ANOVA with correction for multiple comparisons if more than 2 groups were compared, or 2-sided Mann-Whitney *U* test was used when 2 groups were compared (**C**–**E**). Exact *P* values are provided on the graphs.

We investigated the persistence of increased plasma SPP1, S100A12, GAS6, and PROS1 into the SARS-CoV-2^–^ post–COVID-19 phase, often characterized by complex pathologies (26). We compared plasma concentrations in 41 post–COVID-19 patients attending outpatient clinic at (mean ± SEM) 68.60 ± 4.36 days after discharge (Figure 4A), 26 of whom had plasma samples that were available from the peak of acute COVID-19. Longitudinal comparison showed that plasma levels of SPP1, S100A12, and GAS6 were significantly lower in convalescence compared with peak disease, but PROS1 was unchanged (Figure 4B). However, although reduced, the levels of SPP1 and S100A12 remained significantly higher than in healthy control donors, irrespective of whether the prior disease trajectory of COVID-19 was mild/moderate or severe (Figure 4C). This was in contrast to the levels of sensitive markers of inflammation, including IL-6 and LDH, which normalized in post–COVID-19 to levels of those in healthy donors, consistent with resolved acute inflammation (Figure 4D and Supplemental Table 1). GAS6, lower than normal control levels in mild/moderate COVID-19 on hospital admission (Figure 3B), remained low after COVID-19 (Figure 4C).

Most (36 of 41) convalescent patients reported persistence of at least 1 post–COVID-19 symptom (fatigue, musculoskeletal, or respiratory). Increased levels of SPP1 and S100A12 persisted in all patients, although extremely high concentrations SPP1 were restricted to symptomatic patients (Figure 4E). Stratification of patients by symptoms showed that SPP1 and S100A12 levels remained increased and GAS6 decreased in convalescent COVID-19 patients, irrespective of symptom category (Supplemental Figure 8).

Together, these data suggest that the SPP1 and S100A12 myeloid cell inflammatory signature persists after resolution of SARS-CoV-2 infection and may contribute to the pathogenesis of long–COVID-19 syndrome (26).

### SPP1 protein is expressed by COVID-19 BALF macrophages, drives proinflammatory activation of classical monocytes, and the differentiation of neutrophils toward a proinflammatory CD274^+^ (PD-L1^+^) phenotype.

**Figure 5:**
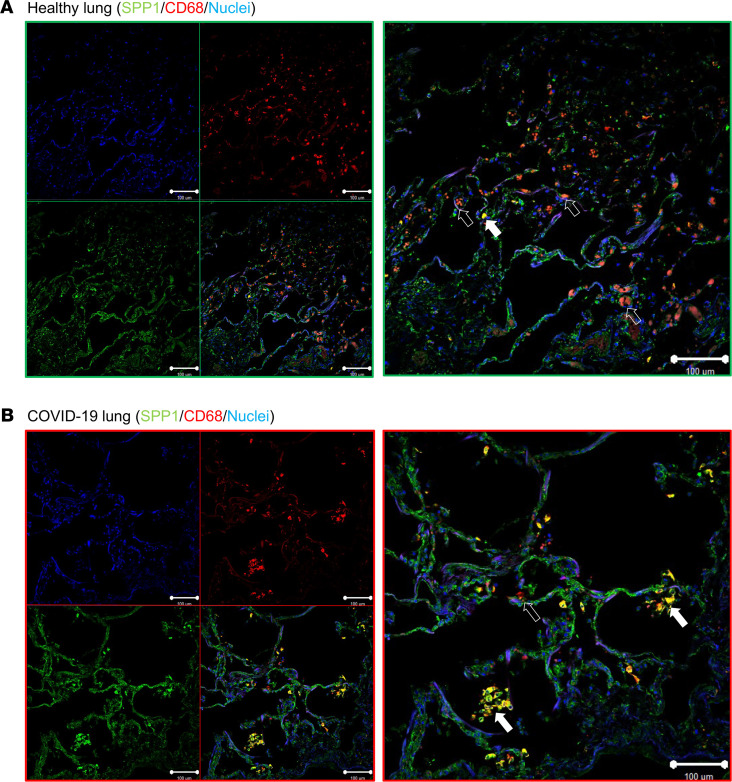
SPP1 protein is expressed in COVID-19 lung by macrophages but not healthy alveolar macrophages. (**A**) Representative immunofluorescence staining of normal lung (*n =* 3) showing SPP1^–^ alveolar macrophage (CD68^+^). (**B**) Representative immunofluorescence staining of COVID-19 lung (*n =* 2) showing SPP1^+^ macrophages (CD68^+^SPP1^+^) in alveoli. Solid white arrows indicate macrophages double-positive for SPP1 and CD68; hollow arrows indicate CD68^+^ and SPP1^–^ macrophages.

**Figure 6:**
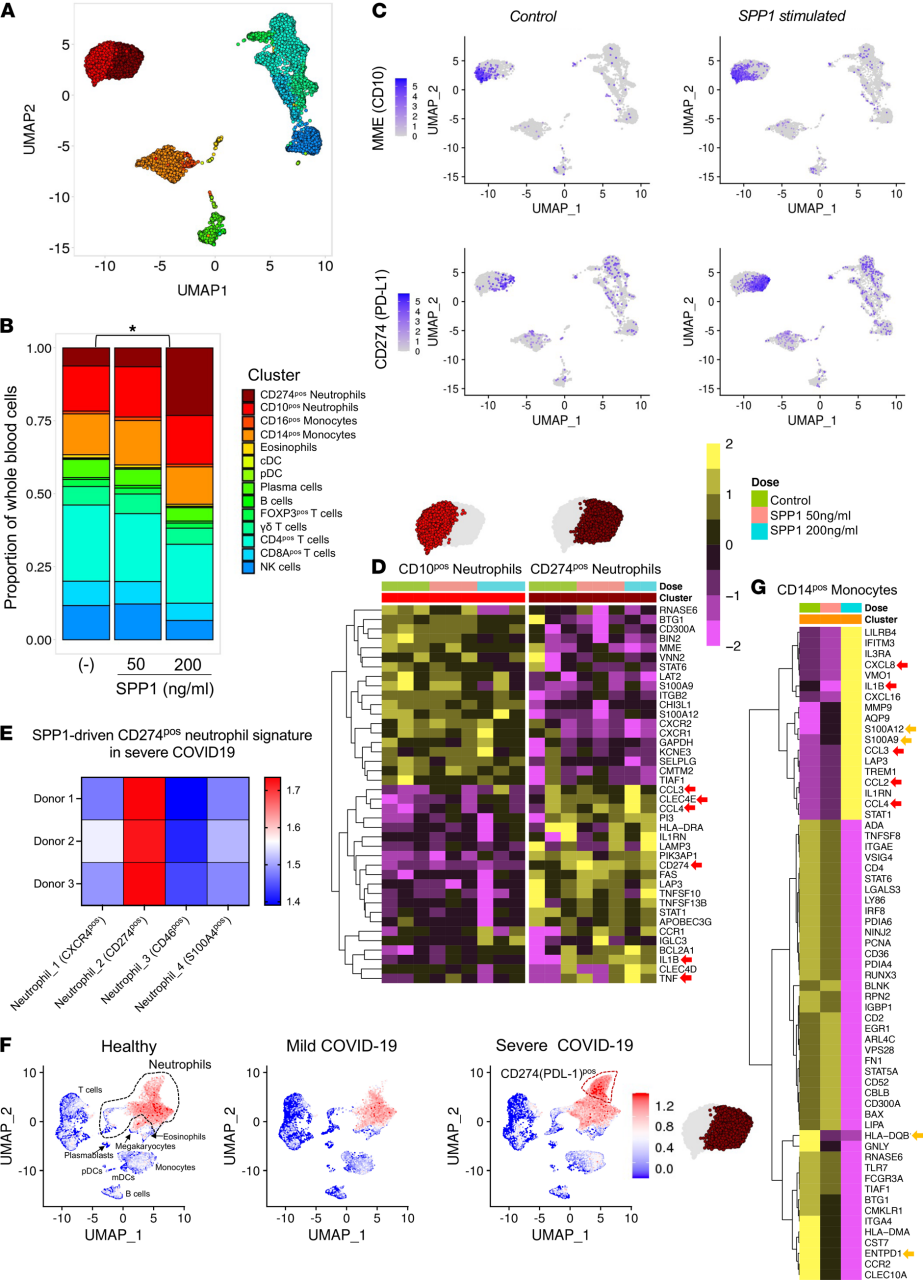
SPP1 stimulation drives proinflammatory CD14^+^ monocyte and CD274^+^ (PD-L1^+^) neutrophil phenotypes. Whole blood cells from healthy donors (*n =* 3) were stimulated with SPP1 (50 or 200 ng/mL) or were unstimulated, for 16 hours. (**A**) UMAP of 13,580 integrated control and SPP1-stimulated blood cells colored by cluster identity. (**B**) Stacked bar plot illustrating cluster proportion of total whole blood cells per condition/dose of SPP1. * *P* < 0.05, 1-way ANOVA with correction for multiple comparisons. (-), unstimulated control. (**C**) UMAPs illustrating change in expression of MME (CD10) and CD274 (PD-L1) by neutrophil clusters following SPP1 stimulation. (**D**) Heatmap showing expression of the top 20 marker genes of each neutrophil phenotype, illustrated as average expression of each gene per sample per condition. Adjusted *P* < 0.05, MAST with Bonferroni correction. (**E**) Average of SPP1-driven CD274^+^ neutrophil gene module score by neutrophil clusters of COVID-19 whole blood cell data set (22). (**F**) UMAP expression of the SPP1-driven CD274^+^ neutrophil gene module in COVID-19 whole blood data set. (**G**) Heatmap showing expression of genes significantly differentially expressed in CD14^+^ monocytes comparing SPP1 (200 ng/mL) stimulation with unstimulated control, illustrated as average expression of gene per condition. Adjusted *P* < 0.05, MAST with Bonferroni correction. Red and orange arrows in **D** and **G** represent genes regulated by 200 ng/mL only, or by 200 and 50 ng/mL SPP1, respectively.

High plasma SPP1 selective for severe COVID-19 (Figure 3B) suggested a role for SPP1 in pathogenesis. SPP1 and CD68 staining of postmortem lung tissue confirmed abundant clusters of SPP1^+^ macrophages in alveoli of COVID-19 patients (*n =* 2), while it was rare in normal lung (*n =* 3; Figure 5, A and B and Supplemental Table 2) and sparse in alveoli of bacterial (*n =* 3) and H1N1 (*n =* 3) pneumonia (Supplemental Figure 9, A and B). To investigate the biological effects of SPP1, we stimulated healthy whole blood cells with SPP1 at concentrations equivalent to those in severe and post–COVID-19 (200 and 50 ng/mL, respectively) (Figure 3B). SPP1 receptors (e.g., CD44/integrins) are expressed by many immune cell types in lung and blood (Supplemental Figure 5 and Supplemental Figure 10); therefore, to capture the effects of SPP1 on all cell types, we used scRNAseq of the whole blood culture (*n =* 3 healthy donors) using the immune gene panel (399 genes) with the BD_Rhapsody system after 16-hour stimulation with SPP1. We sequenced 13,580 cells and identified 14 distinct immune clusters (Figure 6, A and B), including neutrophil, eosinophil, monocyte, DC, NK cell, and lymphocyte cell clusters. The relative proportions of cell clusters revealed that SPP1 stimulation at 200 ng/mL, equivalent to plasma concentration of severe COVID-19, was associated with a significantly increased neutrophil proportion (Figure 6, B and C) with a shift from the normally dominant CD10^+^ (MME^+^) neutrophil phenotype to a CD274^+^ (PD-L1^+^) neutrophil phenotype. The top 20 marker genes of the CD10^+^ and SPP1-differentiated CD274^+^ (PD-L1^+^) neutrophil clusters (Figure 6D and Supplemental Data) revealed that the CD274^+^ (PD-L1^+^) neutrophils represent an aberrantly activated phenotype characterized by high expression of *PD-L1* (*CD274*), which mediates suppression of adaptive immunity, high levels of proinflammatory cytokines/chemokines (e.g., *IL1B, TNF, CCL3*, and *CCL4*), c-type lectins (*CLEC4E/D*) that facilitate neutrophils migration, and the SPP1 receptor *CD44*. Similar aberrant neutrophil activation is a key pathogenic characteristic of severe COVID-19 (51, 52). To investigate if the in vitro SPP1-driven neutrophil cluster transcriptionally replicated any of the neutrophil phenotypes observed in severe COVID-19, we performed unsupervised mapping of the SPP1-driven neutrophil gene module (37 genes; Supplemental Data) into a whole-blood scRNAseq transcriptomic signature of healthy, mild, and severe COVID-19 (22). We identified that enrichment of transcriptional signature of SPP1-driven CD274^+^ (PD-L1^+^) neutrophils was significantly higher in Neutrophil_2 phenotype than any other cell population (Figure 6, E and F) in COVID-19 patients and that this phenotype was uniquely increased in severe disease, suggesting that SPP1 might be responsible for the pathogenic activation of neutrophils in severe COVID-19. This link between SPP1 and neutrophils is supported by the correlation between plasma SPP1 and the blood neutrophils/lymphocytes ratio in our COVID-19 cohort (Figure 3C).

Our analysis also revealed that SPP1 induced the activation of CD14^+^ classical monocytes. At 200 ng/mL, SPP1 strongly increased the expression of alarmins (*S100A12, S100A9*), *IL-1β*, and chemokines (*CXCL8, CCL2-4*) with a commensurate reduction of *MHCII* and antiinflammatory *ENTPD1* (encoding CD39; Figure 6G), which resembles the proinflammatory changes in the blood CD14^+^ monocyte subset in severe COVID-19 (22, 23). SPP1 at post–COVID-19 convalescent plasma concentration (50 ng/mL) did not affect neutrophil activation. However, it increased expression levels of alarmins *S100A12* and *S100A9*, and it decreased the expression of *MHCII* and *ENTPD1* of CD14^+^ monocytes (Figure 6G), suggesting that persistent levels of SPP1 may contribute to long–COVID-19 pathologies by skewing monocytes toward a proinflammatory phenotype. The effect of SPP1 on other cell types was minimal (Supplemental Data). In summary, SPP1 produced by proinflammatory lung-infiltrating macrophages might be an upstream activator of the aberrant innate response in COVID-19 by driving proinflammatory activity of CD14^+^ monocytes and differentiation of CD274^+^ (PD-L1^+^) neutrophils that suppress adaptive immunity and support inflammation.

## Discussion

Our comparative single-cell transcriptomic analysis integrating myeloid cell clusters from COVID-19 pneumonitis and RA synovitis suggested that COVID-19 and RA pathogenesis and resolution might be driven by similar myeloid cell clusters and their signature functional pathways (Supplemental Figure 11). The shared key pathogenic macrophages were SPP1^+^ and S100A12^+^ clusters, and their signature SPP1 and S100A12 mediators were confirmed in COVID-19 cross-sectional and longitudinal patient plasma samples. COVID-19 patients with severe respiratory distress were characterized by an aberrant raised SPP1 and S100A12 cytokine signature that could predict the urgency for ICU transfer and persisted into the post–COVID-19 phase after hospital discharge. An abundance of SPP1^+^ macrophages in the alveolar spaces and high SPP1 plasma levels were unique to severe COVID-19 pneumonia as compared with pneumonias induced by other pathogens. Investigation into SPP1 mechanisms of action revealed that it drives proinflammatory activation of CD14^+^ monocytes and development of PD-L1^+^ pathogenic neutrophils, both hallmarks of severe COVID-19 (1–14). Thus, COVID-19 pneumonitis appears driven by similar pathogenic myeloid cell pathways as those in RA, and their mediators such as SPP1 may be an upstream activator of the aberrant innate response in severe COVID-19 and predictive of disease trajectory including post–COVID-19 monitoring.

In contrast, COVID-19 patients with milder respiratory distress were characterized by increased plasma levels of the inflammation-resolving, antithrombotic cytokine PROS1, which characterized the resident AM FABP4^+^ cluster. This cluster shared transcriptomic similarities with the inflammation-resolving STM TREM2^+^ cluster, suggesting a potential role in controlling lung inflammation.

The BALF FCN1^+^ and FCN1^+^SPP1^+^ macrophage clusters from severe COVID-19 patients shared transcriptomic profiles with STM CD48^hi^S100A12^+^ and CD48^+^SPP1^+^ clusters from active RA, respectively. The functional biology of the STM counterparts (i.e., CD48^hi^S100A12^+^ and CD48^+^SPP1^+^) showed that they were the main producers of pathogenic TNF, IL-6, IL-1β, and chemokines in the synovium of RA, and this resembles the lung hypercytokine environment characterizing severe COVID-19 respiratory distress syndrome (53, 54), suggesting that SPP1^+^ and S100A12^+^ BALF macrophage clusters might be the key source of these mediators in the COVID-19 lung. These shared macrophage clusters of RA/COVID-19 are also characterized by their cluster-unique mediators, SPP1 (CD48^+^SPP1^+^ cluster) and S100A12 (CD48^hi^S100A12^+^cluster; ref. 19). These mediators were upregulated in severe compared with mild/moderate COVID-19, and their high plasma concentrations correlated with respiratory insufficiency and was predictive for urgency of ICU transfer. The increased number of SPP1^+^ macrophages and upregulation of SPP1 plasma levels were selective for severe COVID-19. Many biological functions for SPP1, mostly in tissue remodeling, have been described (39, 55). However, its contribution to COVID-19 pathologies is unclear. We revealed here that SPP1 at the level detected in patients with severe COVID-19 is a potent driver of pathogenic PD-L1^+^ neutrophils that are characteristic of severe COVID-19 (22). Thus, SPP1 might be an upstream regulator of neutrophil-mediated tissue damage and immune-thrombosis that are key pathogenic features of severe COVID-19 (51, 52). In addition, SPP1 induced strong proinflammatory activation of CD14^+^ monocytes, including the expression of S100A alarmins, suggesting that it may contribute to the development of S100A12^+^ macrophage clusters in the lung.

There is an increasing recognition of a syndrome of persistent debilitating symptoms after the COVID-19 patients become negative for SARS-CoV-2 (post–COVID-19). The symptom patterns are complex, but dyspnoea and fatigue are prominent (26). The pathogenic mechanisms of this syndrome are unknown. Emerging transcriptomic evidence shows that changes in blood myeloid cells elicited during acute infection can normalize within 14 days of becoming negative for SARS2-CoV-2 (56). However, some changes can persist for at least 12 weeks, including high levels of surface molecules that regulate monocyte migration into tissue — e.g., *CXCR6* and *VLA*-4 (57). We found that — in contrast, for example, to IL-6 — plasma SPP1 and S100A12 remained significantly higher than normal for at least 10 weeks after infection clearance in the post–COVID-19 convalescent phase. Our study revealed that SPP1, at the concentration detected in the post–COVID-19 stage, induced some of the features of proinflammatory CD14^+^ monocytes — for example, an increase in alarmins (*S100A12* and *S100A9*) and a decrease in *MHCII* molecules. This suggests that a sustained raised level of SPP1 may contribute to post–COVID-19 pathologies and may influence future responses against pathogens by skewing monocytes toward proinflammatory phenotype. At this stage of understanding, it remains unclear whether the plasma SPP1 and S100A12 exclusively originate from the pathogenic BALF clusters persisting in the lung of these patients. However, regardless of SPP1 and S100A12 source, their wide range of proinflammatory and fibrotic functions may be responsible for the respiratory and musculoskeletal symptoms that persist in convalescence.

One of the aims of this study was to apply our recent discovery of inflammation-resolving macrophage pathways in RA to investigate potentially similar inflammation-resolving pathways for COVID-19. We found that tissue-resident lung alveolar FABP4^+^ macrophages and homeostatic synovial lining layer TREM2^+^ macrophages share transcriptomic profiles. This may reflect their homeostatic functions in their respective tissues. In the synovium, the TREM2^+^ macrophages form a lining-layer producing and recycling lubricant synovial fluid, which facilitates joint movement (58). While in lung alveoli, the FABP4^+^ macrophages contribute to alveolar integrity, the recycling of surfactants to maintain patency, and the facilitation of gas exchange (33). One of the pathways they shared was inflammation-resolving TAM (TYRO, AXL, MerTK) receptor pathways. TAM pathways are widely expressed by resident macrophages in many tissues (59, 60), and their deregulation has proinflammatory/autoimmune consequences in preclinical animal models (61, 62) and in patients with RA (19). The scRNAseq data show that, in healthy human BALF, the FABP4^+^ macrophages express the TAM receptor *AXL* and the ligand for the TAM receptor MerTK (*PROS1*) and that these were profoundly repressed in severe COVID-19. Consistent with scRNAseq data, plasma Protein S levels were higher in COVID-19 patients who maintained lung functions (i.e., sustained PaO_2_/FiO_2_ > 200), suggesting a potential role in counterbalancing the severity of inflammation of SARS-CoV-2 infection.

Limitations of the study included the younger age and fewer numbers of subjects within the healthy control group used as a comparator of some of the COVID-19 analysis. These limitations were mitigated by findings that SPP1, S100A12, and PROS1 were unaffected by age. In addition, the control group for the key mediator SPP1 in severe COVID-19 was the non–SARS-CoV-2 pneumonia group that matched COVID-19 for age, and it showed lower levels of SPP1 compared with COVID-19, supporting the link between SPP1 and COVID-19 pathogenesis.

In summary, this study suggests that the pathogenesis of acute severe COVID-19 pneumonitis and RA synovitis might be driven by similar pathogenic myeloid cell clusters/pathways, producing SPP1 that persists into post-COVID-19 syndrome. Further functional studies on identification of common tissue factors driving the differentiation of these pathogenetic macrophage clusters and better understanding the functional interaction between them and the lung environment could clarify mechanisms of pneumonitis and provide evidence for potential repurposing of current antiinflammatory/antifibrotic treatments for COVID-19 (63, 64). Promising data from current COVID-19 clinical trials of drugs already used for the treatment of RA (e.g., dexamethasone, tocilizumab, and baricitinib; refs. 35, 65–69) further support the concept of common pathogenic and resolution mechanisms that could be capitalized upon for COVID-19 therapeutic exploitation.

## Methods

### Data acquisition and analysis

#### Comparison and integration of BALF and ST myeloid cell scRNAseq data.

BALF data were acquired as CellRanger output (GEO, GSE145926) and ST data were acquired from EMBL-EBI:*E-MTAB-8322*. COVID-19 blood and PBMC data (22) were from https://beta.fastgenomics.org/p/schulte-schrepping_covid19

### Quality checks, filtering, clustering of 10X Genomics data sets

#### Seurat (3.1.2) in R created an object (CreateSeuratObject).

Cell-filtering involved removal of cells with < 500 expressed genes (subset, subset = nFeatures_RNA > 500), with set-thresholds for gene-expression level, including mitochondrial genes (percent.mt) to exclude doublets and dying cells. Data normalized using Seurat’s NormalizeData function. Myeloid cells were filtered for expression of CD14, MARCO, and LYZ with the subset function. The top 2000 variable genes were identified using the FindVariableFeatures function. Sample integration of each data set followed the Seurat vignette, integrating all genes common between samples of each condition, using FindIntegrationAnchors and IntegrateData functions (features.to.integrate to normalize all common genes).

#### Individual clustering and dimensional reduction.

UMAP based on PCA cell embeddings were generated by Seurat for each data set using RNA assay. To visualize ST data, the first 12 principal components were used (RunUMAP). These principal components determined the k-nearest neighbors for each cell during shared nearest neighbor (SNN) graph construction before clustering at a resolution of 0.5 (FindNeighbors, FindClusters) and identifying the populations as described (19). In comparison, the first 50 principal components (0.8 resolution) were used for visualization and clustering of BALF data (24), and populations were identified by merging clusters based on expression of FCN1, SPP1, and FABP4. The relative proportion of clusters between conditions was analyzed using Kruskal-Wallis test with Dunn’s correction (Prism v8.4.2).

#### Differential expression analysis.

The Seurat FindAllMarkers function identified cluster markers in individual data sets. The “test.use” function determined genes differentially expressed between clusters within each data set using MAST. As recommended, for DE comparison the nonbatch normalized counts were used. Cluster markers must be expressed by ≥ 40% cells in the cluster (“min.pct” parameter 0.4). Default values were used for all other parameters. Genes are considered significantly DE if *P <* 0.05 (Bonferroni correction, multiplied by number of tests/clusters).

#### Ligand-receptor expression analysis.

CellTalkDB (70) identified putative receptors of mediators of interest (S100A12, SPP1, GAS6, and PROS1). An object containing all sequenced BALF cell types was prepared for visualization of expression of mediators and identified receptors by distinct BALF immune and epithelial clusters. The same quality control (QC) and integrative clustering procedure were followed as described above for analysis of myeloid cells, without filtering cells for expression of CD14, MARCO, and LYZ. The first 30 principal components (0.3 resolution) were used for classification of BALF immune and epithelial cell types (24). Clusters were annotated by marker gene expression, and expression of genes of interest were visualized (FeaturePlot, RNA assay). Pseudobulk expression values of genes of interest were generated per sample, per cell type, and were exported for analysis (GraphPad Prism 9.1.0). A similar strategy was used to analyze SPP1 receptor expression in COVID-19 blood (22), and data were obtained as a Seurat object with clustering and annotation as described above (individual clustering and dimensional reduction).

### Data set comparison

Data set integration was performed as described above for 8993 common genes between ST and BALF. These “integrated” batch-corrected values were set as the default assay, and gene expression values are scaled before principal component analysis (PCA).

#### Clustering and dimensional reduction.

To prevent bias in clustering, we matched BALF and ST cell numbers at 32,139 random cells. Data were rescaled (ScaleData), and the first 30 principal components were visualized by UMAP. Cells colored by original identity illustrated how clusters of each data set overlay.

#### Comparative analyses.

A count matrix with the average expression of all common genes by each cluster was generated (AverageExpression) and hierarchical clustering (dist, hclust) performed. Data were visualized by a dendrogram. PCA was performed on this pseudobulk expression matrix (prcomp). These analyses identified similar clusters between BALF and ST. Shared marker genes between similar clusters were identified by Venny (2.1). To visualize pseudobulk expression of overlapping marker genes, the pheatmap (1.0.12) package visualized shared genes as a heatmap using a custom script. Between–data set comparison of pseudobulk of expression marker genes (unique and common to all clusters) was performed by Pearson correlation (ggscatter, add = “reg.line”, conf.int = TRUE, cor.coef = TRUE, cor.method = “pearson”) performed on the 916, the 721, and the 986 unique and common genes of BALF FCN1^+^/ST CD48^hi^S100A12^+^, BALF FCN1^+^SPP1^+^/ST CD48^+^SPP1^+^, and BALF FABP4^+^/ST TREM2^hi^ clusters, respectively.

### Patients and clinical assessment

#### COVID-19 pneumonia patients.

Reverse transcription PCR (RT-PCR) positive for SARS-CoV-2 by nasopharyngeal swab (NPS) were enrolled (COVID-Hospital, Fondazione Policlinico Universitario Agostino Gemelli, Rome, Italy). Each enrolled patient provided peripheral blood samples and COVID-19 severity was classified on hospital admission and/or before antiinflammatory treatment. Mild/moderate COVID-19 pneumonia was defined based on (mild) normal O_2_ saturation (>94%), or (moderate) abnormal O_2_ saturation (<94%); pneumonia based on imaging (chest x-ray or CT scan); and arterial oxygen partial pressure (PaO_2_ in mmHg) to fractional inspired oxygen (FiO_2_) PaO_2_/FiO_2_ (>200). Severe COVID-19 pneumonia was defined based on abnormal O_2_ saturation (<94%), presence of pneumonia on imaging (x-ray or CT scan), and PaO_2_/FiO_2_ (≤200), or the use of high-flow nasal cannula (HFNC), nonrebreather mask (NRB), bilevel positive airway pressure (BIPAP) or mechanical ventilation and vasopressor drugs use, creatinine clearance greater than 30, and on alanine aminotransferase (ALT) less than 5× the upper limit of normal. Concomitant comorbidities including arterial hypertension, diabetes mellitus, ischemic cardiopathy, COPD, dyslipidaemia, and cancer were recorded. The subsequent need and date of ICU admission, intubation status, and O_2_ therapy for each patient was recorded. Patients were treated following the internal protocol defined by the Gemelli Against COVID-19 task force as described (35).

#### Convalescent post–COVID-19 patients.

Follow-up outpatient attendance (Fondazione Policlinico Universitario Agostino Gemelli) was offered to all discharged COVID-19 patients. Those recruited attended 68.6 ± 4.4 days (mean ± SEM) after discharge and were RT-PCR–negative for SARS-CoV-2. They were assessed by a multidisciplinary team who collected demographic and clinical data, collected blood samples, and performed physical examinations. Persistence of symptoms was recorded by a questionnaire.

#### Patients with community-acquired pneumonia.

Patients with pneumonia, RT-PCR–negative for SARS-CoV-2 by NPS, were enrolled at the Emergency Room (Fondazione Policlinico Universitario Agostino Gemelli). Demographic and clinical parameters were recorded, and blood samples were collected. These formed a comparison pathological control group for COVID-19 pneumonia. All patients and healthy control demographics and clinical characteristics are summarized in Supplemental Table 1.

#### Lung tissue IHC for SPP1^+^ macrophages.

Postmortem lung tissue was obtained from COVID-19 pneumonia (*n =* 2), bacterial pneumoniae (*n =* 3), H1N1 pneumonia (*n =* 3), and normal donors (*n =* 3). Demographic and clinical data are summarized in Supplemental Table 2. Autopsies were performed at the Department of Woman and Child Health and Public Health, Institute of Pathology of The Fondazione Policlinico Universitario Agostino Gemelli, in accordance with the guidelines of the Royal College of Pathologists (www.rcpath.org). Tissue collection was performed in accordance with appropriate protocols (71). Lung tissue was fixed in 10% neutral-buffered formalin, embedded in paraffin, sectioned at 4 mm, and stained as described (19). Briefly, after blocking (2 hours, 10% human serum, 10% of goat serum, 1% BSA in TBS; Thermo Fisher Scientific) at room temperature, sections were incubated overnight at 4°C with the primary rabbit anti–human osteopontin (SPP1) antibody (Abcam, ab8448, 1:100) and mouse anti–human CD68 (Dako, clone PG-M1, 1:40) in blocking buffer above. Sections were washed twice (5 minutes, TBS plus 0.025% Triton X-100). Secondary antibody goat anti-rabbit IgG Alexa Fluor 488 (1:100), and goat anti–mouse IgG Alexa Fluor 660 (1:100) (both from Invitrogen) were incubated in dilution buffer (TBS with 1% BSA) and incubated for 1 hour at room temperature. Sections were washed 3 times for 5 minutes in TBS and counterstained with DAPI (H-1800-2/VECTASHIELD Vibrance Antifade Mounting Medium with DAPI), visualized with confocal microscopy with Zeiss Zen Black software. Negative controls were performed throughout using isotype matched antibodies.

#### SPP1, S100A12, GAS6, PROS1, IL-6, IL-8, IL-1β, and TNF-α ELISA.

Blood samples were centrifuged (600*g*/15 minutes) and plasma aliquots were stored at –80°C until analysis by ELISA for SPP1 (BMS2066; Thermo Fisher Scientific), S100A12 (DY1052; R&D Systems), GAS6 (DY885B; R&D Systems), and PROS1 (NBP2-60585; NOVUS Biological). IL-6, IL-8, TNF-α and IL-1β plasma levels were quantified by single-plex ELISA (Multi-cytokine test for ELLA, Bio-Techne).

#### SPP1 stimulation of whole blood for scRNAseq (BDRhapsody) analysis.

Leukocytes were collected by centrifugation from heparinized blood samples (30 mL from 3 healthy donors) after RBC lysis (ACK Lysing Buffer, A1049201, Thermo Fischer Scientific). Leukocytes were plated at 5 × 10^6^/well in a 24-well cell-culture plate in 0.5 mL of RPMI 1640 compete medium containing SPP1 (PeproTech, 120-035) at concentrations of 0, 50, and 200 ng/mL. After 16 hours, cells were deattached (Accutase solution; A6964, Merck), transferred to U-bottom 96-well plates, and harvested by centrifugation at 200*g* for 4 minutes at 4°C. Cells from each donor/condition were labeled with unique Tags (below) using a Single-Cell Multiplexing Kit (BD Biosciences, 633781) for 20 minutes at room temperature according to manufacturer protocol.

Tag 1, ATTCAAGGGCAGCCGCGTCACGATTGGATACGACTGTTGGACCGG; Tag 2, TGGATGGGATAAGTGCGTGATGGACCGAAGGGACCTCGTGGCCGG; Tag 3, CGGCTCGTGCTGCGTCGTCTCAAGTCCAGAAACTCCGTGTATCCT; Tag 4, ATTGGGAGGCTTTCGTACCGCTGCCGCCACCAGGTGATACCCGCT; Tag 5, CTCCCTGGTGTTCAATACCCGATGTGGTGGGCAGAATGTGGCTGG; Tag 6, TTACCCGCAGGAAGACGTATACCCCTCGTGCCAGGCGACCAATGC; Tag 7, TGTCTACGTCGGACCGCAAGAAGTGAGTCAGAGGCTGCACGCTGT; Tag 8, CCCCACCAGGTTGCTTTGTCGGACGAGCCCGCACAGCGCTAGGAT; Tag 9, GTGATCCGCGCAGGCACACATACCGACTCAGATGGGTTGTCCAGG.

Cells were washed ×3 with PBS, with centrifugation (200*g* for 4 minutes at 4°C), after which the tagged culture variants were pooled and loaded onto the scRNAseq BD Rhapsody Cartridge using the BD Rhapsody Cartridge Reagent Kit (catalog 633731) according to the manufacturer’s protocol. Single-cell cDNA was prepared using the BD Rhapsody cDNA Kit (catalog 633773). This was followed by single-cell mRNA and tag-library preparation using BD Rhapsody Targeted mRNA and the Tag Amplification Kit (catalog 633774) and primers for the BD Rhapsody Immune Response Panel (399 genes; catalog 633750). Libraries were sequenced at a depth of 1,083,775 ± 236,302 (mean ± SEM) reads per tag using Illumina NextSeq 500 (Glasgow Polyomics). Then, 1535 ± 383 cells (mean ± SEM) per tag were successfully sequenced. For analysis, the sequencing reads were processed with BD Genomics Rhapsody Analysis Pipeline CWL v.1.0 using the Seven Bridges platform. The Seurat package (3.1.5) in R was used to create an object from the RSEC_MolsPerCell.csv output file for each sample tag (CreateSeuratObject). Following the standard analysis protocol (19), normalization and data scaling were performed, followed by PCA of the top 2000 variable genes (RunPCA). A UMAP plot (RunUMAP) was generated from the first 30 principal components. The same principal components were used to determine k-nearest neighbors for each cell during SNN graph construction, before clustering at a chosen resolution of 1.2 (FindNeighbors, FindClusters). Differential expression was performed (FindAllMarkers, test.use = MAST) to identify cluster markers and variable genes between SPP1 doses. Genes are considered significantly DE if *P <* 0.05 with Bonferroni correction (Supplemental Data). To visualize heatmaps, the pheatmap (1.0.12) package was adapted. The normalized expression values were used to perform pseudobulk expression analysis of each sample (AverageExpression). Raw data are accessible at EMBL-EBI (*E-MTAB-10430*). Using Seurat (AddModuleScore), we scored the clusters of BDRhapsody COVID-19 whole-blood data set (22) based on average expression of 37 marker genes that characterized the SPP1-driven CD274^+^ neutrophil phenotype. A positive score suggests that this module of genes is expressed more highly in a particular cell than expected across the general population, described in more detail in ref. 72. The module score was used to create a new assay for visualization and was illustrated using UMAP expression and pseudobulk sample expression heatmap.

### Statistics

The scRNAseq data comparison is described in data comparison and pathway analysis. Statistical analysis of patient clinical, demographic, and plasma cytokine levels were performed using SPSS V.20.0 (SPSS) and GraphPad Prism software version 9.0.0. Categorical and quantitative variables were described as frequencies, percentage, median with the interquartile range, or mean ± SEM. Data on demographic and clinical parameters were compared between patients by Mann-Whitney *U* test or χ^2^ test, as appropriate. Cytokine concentrations between multiple patient groups and healthy donors were compared using 1-way ANOVA (or Kruskal-Wallis) with Dunn’s or Tukey’s correction for multiple comparisons, or 2-sided Mann-Whitney *U* test for 2 groups. Cytokine categories at cut-off level 108 ng/mL, 59 ng/mL, 24 ng/mL, and 15 μg/mL for SPP1, S100A12, GAS6, and PROS1, respectively, were selected based on the median levels in all COVID-19 patients. Linear correlations between cytokines and continuous parameters were performed using the Spearman’s rank test. Kaplan-Meyer analysis estimated the probability of “no need to be transferred to ICU during hospitalization” for COVID-19 patients with acute pneumonia based on the previously determined cut-off plasma values of SPP1, S100A12, GAS6, and PROS1 at the time of hospital admission. *P* < 0.05 was considered as statistically significant. Exact *P* values are provided on graphs.

### Study approval

The study was approved by the Committee of the Fondazione Policlinico Universitario Agostino Gemelli IRCCS — Università Cattolica del Sacro Cuore (nos. 12401/20 and 0024184/20) and University of Glasgow, MVLS College Ethical Committee (no. 2012073).

## Author contributions

MKS oversaw the project and, with SA, interpreted clinical results and, with LM, interpreted computational and experimental results. MKS, LM, SA, and CM wrote the manuscript with feedback from all authors. LM analyzed and integrated 10X Genomics scRNAseq data and interpreted all computational data. SA and BT evaluated cytokine levels in patients’ samples and performed statistical analysis with clinical data integration. LM and MKS performed SPP1 stimulation of whole blood and subsequent scRNAseq and data analysis. DS performed BD_Rhapsody sequencing library preparation and pathway analysis on scRNAseq data. LM analyzed BD_Rhapsody SPP1 data, and with DS mapped to COVID-19 data sets. TDO and MKS supervised all computational analysis in the study. BT, M. Sali, and M. Sanguinetti organized COVID-19 samples collection and handling and performed ELISA with ELLA technology. SP, AP, LP, A. Cingolani, RM, MF, MA, FL, FF, AG, A. Carfì, and EG provided clinical management of patient cohorts during acute phase hospitalization and at the postacute outpatient clinic and provided longitudinal clinical data. ES and VA provided lung tissues from cadavers and provided clinical data. AE assisted with ELISAs and preparation of tissue samples. IBM and EG assisted with running the project. EG conceived the COVID-19 translational research activity for the “Immunology Core facility” of the Fondazione Policlinico Universitario Agostino Gemelli IRCCS. Our study is a collaboration between 2 centers. The first 2 and last 2 authors contributed equally. With agreement between all authors, and reflection on each author’s contribution, we alternated the first and last authors as listed to accurately reflect the collaborative effort of the 2 centers.

## Supplementary Material

Supplemental data

Supplemental Data Set 1

## Version 1 06/18/2021

In-Press Preview

## Version 2 07/08/2021

Electronic publication
