# Supplementary material for: COVID-19 and RA share an SPP1 myeloid pathway that drives PD-L1+ neutrophils and CD14+ monocytes
Source: JCI Insight. 2021 Jun 18;6(13):e147413. doi: 10.1172/jci.insight.147413 (PMC8328085; doi:10.1172/jci.insight.147413)
Supplement: Supplemental data [file jciinsight-6-147413-s190.pdf]

Supplementary Material

Title: COVID-19 and RA share SPP1 myeloid pathway that drives PD-L1^{pos} neutrophils and CD14^{pos} monocytes

Authors: [1]†Lucy MacDonald & [1,2,3]†Stefano Alivernini, [1,3]Barbara Tolusso, [1]Aziza Elmesmari, [1]Domenico Somma, [2]Simone Perniola, [2]Annamaria Paglionico, [2]Luca Petricca, [2]Silvia Laura Bosello, [4]Angelo Carfì, [5,6]Michela Sali, [7]Egidio Stigliano, [8,9]Antonella Cingolani, [8,9]Rita Murri, [7]Vincenzo Arena, [8,9]Massimo Fantoni, [10,11]Massimo Antonelli, [4]Francesco Landi, [12,13]Francesco Franceschi, [5,6]Maurizio Sanguinetti, [1]Iain B. McInnes, [14]Charles McSharry, [15,16]Antonio Gasbarrini, [1,]*Thomas D. Otto, [1]*^Mariola Kurowska-Stolarska & [1,2,3]*^Elisa Gremese

Supplementary Figure Legend

Supplementary Figure 1. Illustration of COVID-19 BALF (GSE145926) and RA synovial tissue macrophages (E-MTAB-8322) data sets. (**A**) UMAP of 52,380 BALF macrophages (ref 24) using Seurat _v3.1. Each dot represents one cell and is coloured by cluster identity. (**B**) Box-and-whisker plots illustrating BALF macrophage cluster proportions comparing healthy donors with mild and severe COVID-19. (**C**) UMAP of 32,139 synovial tissue (ST) macrophages (ref 19) using Seurat _v3.1. Each dot represents one cell and is coloured by cluster identity. (**D**) Box-and-whisker plots illustrating ST macrophage cluster proportions between conditions. Two-way Anova with Tukey's correction for multiple comparison, or **&**Two-sided Mann-Whitney for two-group comparison, exact p-values provided on the graphs.

Supplementary Figure 2. Expression of *S100A12, SPP1, GAS6* and *PROS1* in COVID-19 BALF dataset (ref 24). (**A**) UMAP of 66,852 cells from bronchoalveolar lavage fluid (BALF) of healthy controls, and patients with mild and severe COVID-19. Cells are colored by cell

type identity. (**B**) UMAPs illustrating expression of *S100A12*, *SPP1*, *GAS6* and *PROS1* in BALF immune and epithelial cell populations across healthy, mild and severe COVID-19. (**C**) Bar plots illustrating normalized expression values of common mediators *S100A12*, *SPP1*, *GAS6* and *PROS1* per cell across all BALF immune and epithelial cell types. Each dot represents the average expression of these markers per cell type and per sample. Bar height is the mean value with error bars illustrating standard deviation. Healthy (n=3), mild COVID-19 (n=3) and severe COVID-19 (n=6).

Supplementary Figure 3. Expression of identified receptors for GAS6 and PROS1 in COVID-19 BALF dataset (ref 24). (**A**) UMAP of 66,852 cells from bronchoalveolar lavage fluid (BALF) of healthy controls, and patients with mild and severe COVID19. Cells are colored by cell type identity. (**B**) Ligand-Receptor pairs for GAS6 and PROS1 identified using CellTalkDB. (**C**) Heatmap illustrating average expression by each cell type, per condition of GAS6/PROS1 shared receptors. (**D**) UMAPs illustrating expression of GAS6/PROS1 shared receptors in BALF immune and epithelial cell populations across healthy, mild and severe COVID-19.

Supplementary Figure 4. Distribution of plasma levels of SPP1, S100A12, GAS6 and PROS1 in COVID-19 patients. (**A**) Levels of mediators in patients with acute COVID-19 pneumonia (n=92), patients negative for SARS-CoV-2 with community acquired pneumonia (n=29), COVID-19 convalescent patients (n=41) and healthy controls (n=10). (**B**) The combination of cut off values for SPP1, S100A12, GAS6 and PROS1 that could define the % of patients with COVID-19 pneumonia and respiratory failure ($PaO_2/FiO_2 \leq 200$). (**C**) Plasma levels of mediators in patients with acute COVID-19 pneumonia (n=92), patients negative for

SARS-CoV2 with community acquired pneumonia (n=29) and COVID-19 convalescent patients (n=41) stratified based on age category. (**D**) % of patients with COVID-19 acute pneumonia (n=92) having high SPP1 (≥108 ng/ml), S100A12 (≥59 ng/ml), GAS6 (≥24 ng/ml) and PROS1 (≥15 μg/ml) plasma levels in different age categories. (**E**) % of female and male patients with COVID-19 acute pneumonia (n=92) stratified based on having high SPP1 (≥108 ng/ml), S100A12 (≥59 ng/ml), GAS6 (≥24 ng/ml) and PROS1 (≥15 μg/ml) plasma levels. *One-way ANOVA (Kruskal-Wallis test) with Dunn's correction for multiple comparisons or two-sided Mann-Whitney was used, exact p-values are provided on the graphs.

Supplementary Figure 5. Expression of identified receptors for S100A12 and SPP1 in COVID-19 BALF dataset (ref 24). (**A**) UMAP of 66,852 cells from bronchoalveolar lavage fluid (BALF) of healthy controls, and patients with mild and severe COVID-19. Cells are colored by cell type identity. (**B**) Ligand-Receptor pairs for S100A12 identified using CellTalkDB. (**C**) Heatmap illustrating average expression by each cell type, per condition of S100A12 receptors. (**D**) Ligand-Receptor pairs for SPP1 identified using CellTalkDB. (**E**) Heatmap illustrating average expression of SPP1 receptors by each cell clusters, per condition. (**F**) UMAPs illustrating expression of SPP1 receptors in BALF immune and epithelial cell populations across healthy, mild and severe COVID-19.

Supplementary Figure 6. Levels of SPP1, S100A12, GAS6 and PROS1 in COVID-19 patients stratified based on pharmacological treatments. SPP1, S100A12, GAS6 and PROS1 were measured in plasma of healthy (n=10) and COVID-19 patients with mild/moderate (n=29) or severe (n=63) disease. * One-way ANOVA (Kruskal-Wallis test)

with Dunn's correction for multiple comparisons or two-sided Mann-Whitney was used, exact p-values are provided on the graphs.

Supplementary Figure 7. Levels of SPP1, S100A12, GAS6 and PROS1 in COVID-19 patients with different comorbidities. Plasma levels of mediators in patients with acute COVID-19 pneumonia (n=92) stratified based on comorbidities (arterial hypertension, diabetes mellitus, dyslipidaemia, ischemic cardiopathy, COPD and neoplasm). Two-sided Mann-Whitney test. Exact p-values provided on the graphs.

Supplementary Figure 8. Distribution of SPP1, S100A12, GAS6 and PROS1 in convalescent COVID-19 patients based on a singular post-COVID-19 symptom. SPP1, S100A12, GAS6 and PROS1 in convalescent COVID-19 patients (n=41) stratified based on experiencing a particular symptom, e.g., fatigue, musculoskeletal or respiratory symptoms persistence. Two-sided Mann-Whitney U test, exact p-values on the graphs.

Supplementary Figure 9. SPP1 expressing macrophages are present in lungs of patients with bacteria and H1N1 induced pneumonias however to a lesser degree compared to patients with COVID-19 (Figure 5). Representative Immunofluorescence staining of lung with bacteria induced pneumonia (**A**) (n=3) and H1N1 induced pneumonia (**B**) (n=3) showing SPP1 positive and negative macrophages ($CD68^{pos}$). White solid arrows indicate double SPP1 and CD68 positive cells; white hollow arrows indicate CD68 positive and SPP1 negative cells**.**

Supplementary Figure 10. Expression of identified receptors for SPP1 in COVID-19 whole blood dataset (ref 22). (**A**) UMAP of 122,954 cells from control, mild and severe

COVID-19 whole blood. (**B**) Proportion plot illustrating the frequency of each cluster per total number of cells and per condition. Severe COVID-19 is dominated by Neutrophil_2. (**C**) UMAPs illustrating expression of SPP1 receptors in whole blood cell populations across healthy, mild and severe COVID-19.

Supplementary Figure 11. COVID-19 and RA share pathogenic and resolving myeloid pathways. A) Bronchoalveolar (BALF) and synovial tissue (ST) macrophages are heterogenous. Healthy lung contains FABP4^{high} homeostatic alveolar macrophages (AM) that maintain efficient gas exchange, and a smaller FABP4^{low}SPP1^{pos} cluster with an as-yet unknown function. During SARS-CoV-2 infection, distinct FCN1^{pos} and FCN^{pos}SPP1^{pos} macrophage clusters emerge in alveoli replacing the homeostatic AM. In healthy joints, predominant TREM2^{high} and TREM2^{low} with a contribution of FOLR2^{pos}LYVE1^{pos} macrophage clusters form the synovial lining layer. The sublining layer is populated by FOLR2^{pos}LYVE1^{pos}, FOLR2^{pos}ID2^{pos}, FOLR2^{pos}ICAM1^{pos} macrophage clusters, and myeloid CLEC10a^{pos}MHCII^{high} dendritic cells. During RA, new CD48^{high}S100A12^{pos} and CD48^{pos}SPP1^{pos} ST macrophage clusters emerge, and these are the main producers of pro-inflammatory mediators and induce pathogenic changes in adjacent stromal tissue. In COVID-19, the BALF FCN1^{pos} and FCN^{pos}SPP1^{pos} macrophage clusters transcriptionally resemble these pathogenic ST CD48^{high}S100A12^{pos} and CD48^{pos}SPP1^{pos} clusters. Disease remission in RA is associated with the regulatory functions of TREM2^{high} and FOLR2^{pos}LYVE1^{pos} macrophage clusters that produce inflammation-resolving mediators (resolvins and IL-10) and induce homeostatic repair in adjacent stromal cells, mediated by MerTK. MerTK is a member of the tyrosine kinase receptor TAM family (TYRO, AXL, MerTK), and ligating TAM receptors by ligands GAS6/PROS1 forms a homeostatic brake on inflammation. Healthy alveolar FABP4^{pos} macrophages transcriptionally resemble arthritis resolving TREM2^{high}

macrophages, suggesting potentially similar homeostatic mechanisms. These alveolar FABP4^{pos} macrophages constitutively express *AXL* and the MerTK ligand *PROS1*. Expression of these substantially decreases with disease severity. **B)** Pathogenic RA/COVID-19 shared FCN^{pos}SPP1^{pos} cluster localises in alveoli of COVID-19 lung and its key marker, SPP1 is significantly increased in patients with severe disease, positively correlates with respiratory distress and persist into patient' convalescence phase**.** SPP1 drives pro-inflammatory activation of CD14^{pos} monocytes and development of PD-L1^{pos} pathogenic neutrophils, both hallmarks of severe COVID-19. This Figure was prepared using BioRender licence of University of Glasgow.

Supplementary Table 1. Demographic and clinical characteristics of patients with COVID-19 pneumonia, community acquired pneumonia and healthy donors used for the evaluation of SPP1, S100A12, GAS6 and PROS1 plasma levels.

	Healthy controls (n=10)	Normal levels (range)	Community acquired (n=29)	Mild/Moderate COVID19 (n=29)	Severe COVID19 (n=63)	Convalescent COVID-19 (n=41)	*Mild/Moderate vs Severe COVID19*	*Severe COVID-19 vs community pneumonia*	*Mild/Moderate vs community pneumonia*	*Severe vs convalescent*	*Mild/Moderate vs convalescent*	*Community pneumonia vs convalescent*
**Age, years (mean ± SEM)**	47.80 ± 2.81	-	63.86 ±4.23	55.28 ± 3.44	65.79 ± 1.53	59.48 ± 1.97	***0.003***	*0.970*	*0.133*	***0.012***	*0.295*	*0.296*
**Sex, Male n(%)**	5(50.0)	-	16 (55.2)	16 (55.2)	55 (87.3)	35 (85.4)	***0.0006***	***0.0006***	*1.00*	*0.775*	***0.005***	*0.005*
**Arterial hypertension, n(%)**	-	-	11 (37.9)	10 (34.5)	32 (50.8)	18 (43.9)	*0.144*	*0.250*	*0.784*	*0.492*	*0.428*	*0.617*
**Diabetes mellitus, n(%)**	-	-	4 (13.8)	4 (13.8)	14 (22.2)	6 (14.6)	*0.344*	*0.343*	*1.00*	*0.337*	*0.921*	*0.921*
**Dyslipidemia, n(%)**	-	-	6 (20.7)	3 (10.3)	9 (14.3)	5 (14.6)	*0.602*	*0.439*	*0.276*	*0.760*	*0.811*	*0.359*
**Ischemic cardiopathy, n(%)**	-	-	5 (17.2)	0 (0.0)	9 (14.3)	6 (14.6)	*0.032*	*0.713*	***0.0193***	*0.961*	***0.031***	*0.767*
**COPD, n(%)**	-	-	3 (10.3)	2 (6.9)	2 (3.2)	1 (2.4)	*0.416*	*0.158*	*0.640*	*0.827*	*0.364*	*0.160*
**PaO_2/FiO_2, (mean ± SEM)**	-	More than 400	414.82 ± 26.36	415.49 ± 21.77	206.08 ± 14.76	411.88 ± 13.11	***<0.0001***	***<0.0001***	*0.896*	***<0.0001***	*0.755*	*0.887*
**PaO_2/FiO_2<200, n(%)**		**-**	1 (3.4)	2 (6.9)	36 (57.1)	0 (0.0)	***<0.0001***	***<0.0001***	*0.553*	***<0.0001***	*0.088*	*0.231*
**Symptoms duration, days (mean ± SEM)**	-	-	6.48 ± 1.13	7.79 ± 0.98	11.77 ± 0.64	-	***<0.0001***	***<0.0001***	*0.197*	-	-	-
**LDH, UI/L (mean ± SEM)**	-	<250	250.56 ± 25.71	234.00 ± 16.51	362.78 ±23.27	193.88 ± 7.74	***<0.0001***	***<0.0001***	*0.822*	***<0.0001***	*0.109*	*0.278*
**Albumin,g/L (mean ± SEM)**	-	34-48	37.00 ± 1.52	38.82 ± 1.41	30.92 ± 0.73	41.94 ± 0.56	***<0.0001***	***<0.0001***	*0.549*	***<0.0001***	***0.026***	***0.008***
**CRP mg/L, (mean ± SEM)**	-	< 5	73.92 ± 15.33	29.54 ± 7.87	127.76 ±11.35	12.37 ± 5.21	***<0.0001***	***<0.0001***	*0.073*	***<0.0001***	***0.009***	***<0.0001***
**Fibrinogen, mg/dL (mean ± SEM)**	-	200-400	518.81 ± 47.81	431.26 ± 32.64	546.75 ±36.71	312.59 ± 15.84	***0.049***	*0.458*	*0.376*	***<0.0001***	***0.001***	***<0.0001***
**D-dimer, ng/mL (mean ± SEM)**	-	< 500	3143.42 ± 1457.29	1366.48 ± 317.74	4773.91 ±1400.72	768.44 ± 157.23	***0.012***	*0.100*	*0.362*	***<0.0001***	*0.103*	***0.014***
**Haemoglobin, g/L, (mean ± SEM)**	-	12-15	12.86 ± 0.35	13.67 ± 0.35	12.98 ± 0.21	13.57 ± 0.23	*0.190*	*0.711*	*0.169*	*0.128*	*1.000*	*0.135*
**Platelets,x10^9/L (mean ± SEM)**	-	150-450	227.76 ±19.09	264.82 ±19.05	296.77 ±16.08	251.71 ± 13.84	*0.216*	*0.007*	*0.180*	*0.148*	*0.750*	*0.102*
**White blood cells, x10^9/L (mean ± SEM)**	-	4-10	10.89 ± 0.89	6.35 ± 0.48	6.85 ± 0.34	6.42 ± 0.49	*0.486*	***<0.0001***	***<0.0001***	*0.295*	*0.713*	***<0.0001***
**Neutrophils, x10^9/L (mean ± SEM)**	-	2-7	8.55 ± 0.96	4.28 ± 0.42	5.11 ± 0.32	3.92 ± 0.49	*0.148*	***<0.0001***	***<0.0001***	***0.002***	*0.173*	***<0.0001***
**Lymphocytes, x10^9/L (mean ± SEM)**	-	1-3	1.62 ± 0.18	1.61 ± 0.16	1.18 ± 0.13	1.85 ± 0.12	*0.002*	*0.009*	*0.931*	***<0.0001***	*0.059*	*0.170*
**Monocytes, x10^9/L (mean ± SEM)**	-	0.2-1.0	0.46 ± 0.09	0.48 ± 0.23	0.45 ± 0.19	0.19 ± 0.07	*0.774*	*0.283*	*0.325*	***0.029***	*0.132*	*0.018*
**N/L, (mean ± SEM)**	-	-	10.00 ± 2.12	3.18 ± 0.35	6.40 ± 0.49	2.51 ± 0.38	***<0.0001***	*0.950*	***0.004***	***<0.0001***	***0.041***	***<0.0001***
**ICU transfer, n(%)**	-	-	1 (3.4)	0 (0.0)	23 (36.5)	-	***<0.0001***	***<0.0001***	*0.313*	-	-	-
**Death, n(%)**	-	-	2 (6.9)	1 (3.4)	3 (4.8)	-	*0.7741*	*0.8976*	*0.553*	-	-	-

COVID-19: Coronavirus Disease 19; **COPD**: Chronic Obstructive Pulmonary Disease; **PaO_2/FiO_2:** Pressure of Arterial Oxygen to Fractional Inspired Oxygen Concentration ratio (300 - 200 is mild, 200 -100 moderate and <100 severe); **CRP:** C-Reactive protein; **LDH:** Lactate dehydrogenase; **ICU:** Intensive Care Unit; **N/L:** Neutrophils/Lymphocytes ratio.

Supplementary Table 2. Demographic and Clinical characteristics of cadavers whose lung tissue was used for immunohistochemistry.

ID	Gender	Age (years)	Diagnosis
1	F	43	H1N1 pneumonia
2	M	56	H1N1 pneumonia
3	M	53	H1N1 pneumonia
5	M	49	Healthy control
6	M	85	Healthy Control
7	M	89	Healthy control
8	M	69	Bacterial pneumonia
9	M	73	Bacterial pneumonia
10	M	83	Bacterial pneumonia
12	M	86	COVID-19 pneumonia
13	M	59	COVID-19 pneumonia

Supplementary Table 2 legend. M: Male; **F:** Female; **H1N1:** Hemagglutinin Type 1 and Neuraminidase Type 1 Influenza strain; **COVID-19:** Coronavirus disease of 2019.

Supplementary Figure 1

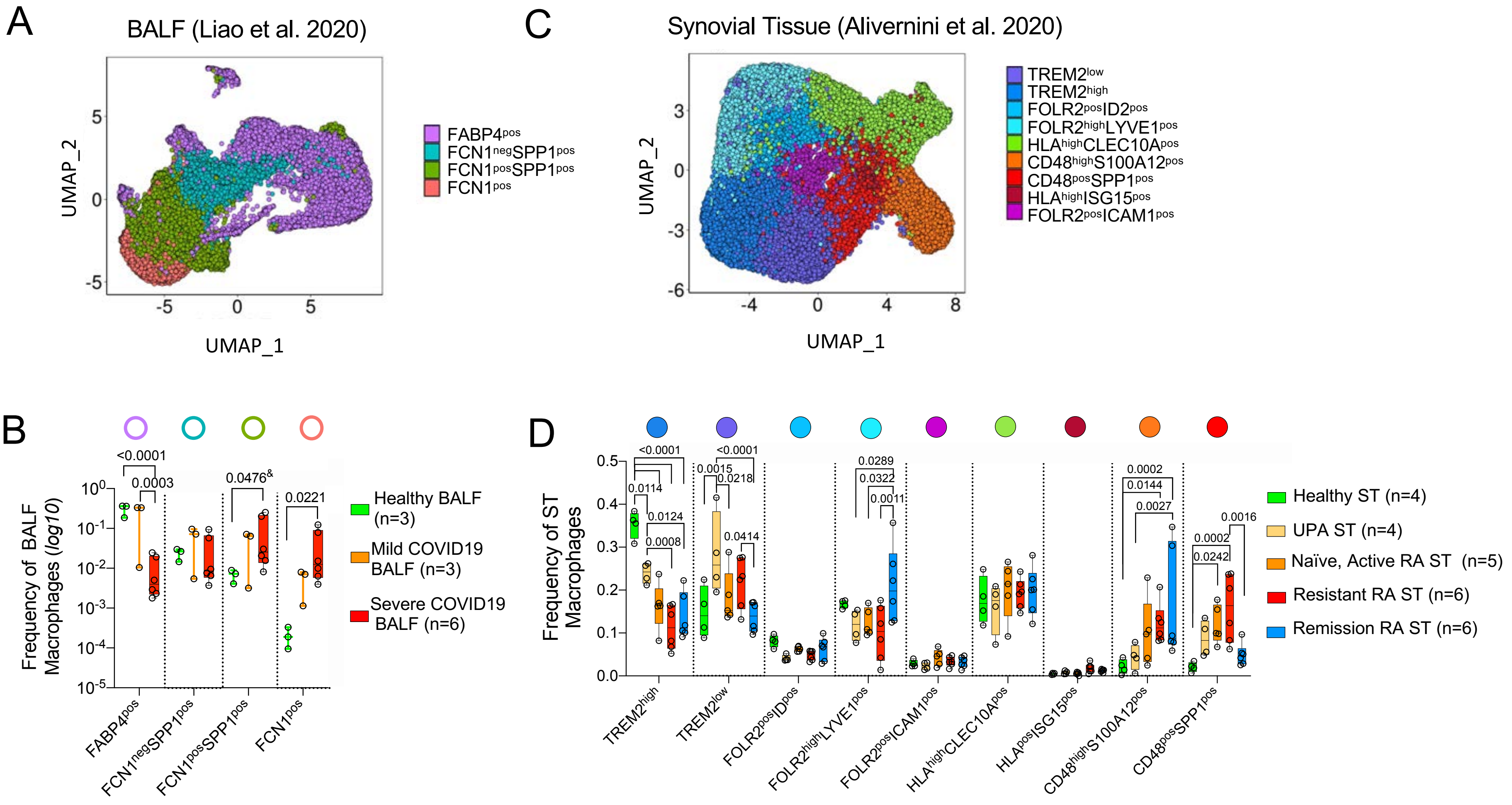

Supplementary Figure 1. Illustration of COVID-19 BALF (GSE145926) and RA synovial tissue macrophages (E-MTAB-8322) data sets. (**A**) UMAP of 52,380 BALF macrophages (ref 24) using Seurat _v3.1. Each dot represents one cell and is colored by cluster identity. (**B**) Box-and-whisker plots illustrating BALF macrophage cluster proportions comparing healthy donors with mild and severe COVID-19. (**C**) UMAP of 32,139 synovial tissue (ST) macrophages (ref 19) using Seurat _v3.1. Each dot represents one cell and is colored by cluster identity. (**D**) Box-and-whisker plots illustrating ST macrophage cluster proportions between conditions. Two-way Anova with Tukey's correction for multiple comparison, or **&**Two-sided Mann-Whitney for two-group comparison, exact p-values provided on the graphs.

Supplementary Figure 2

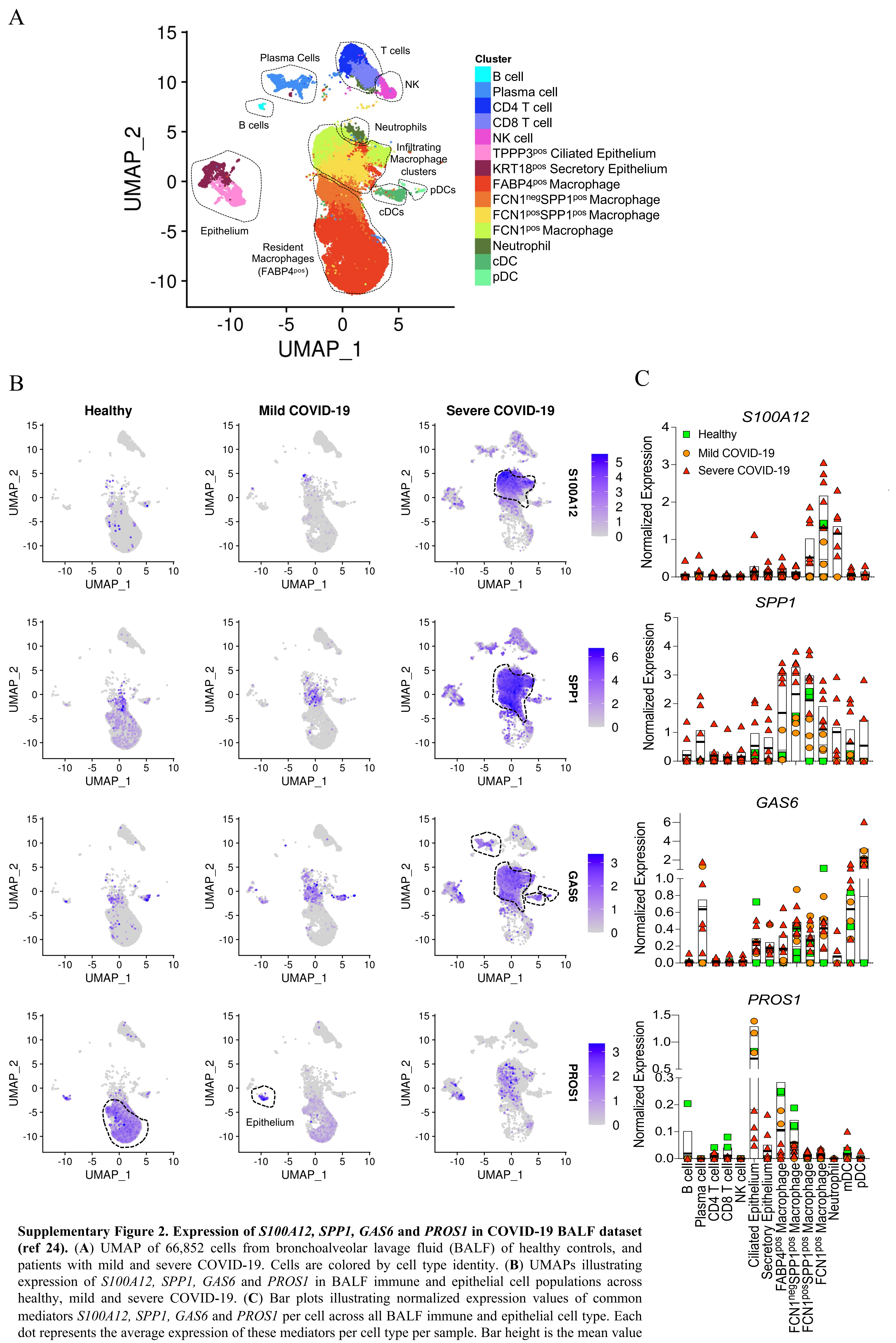

Supplementary Figure 2. Expression of *S100A12, SPP1, GAS6* and *PROS1* in COVID-19 BALF dataset (ref 24). (**A**) UMAP of 66,852 cells from bronchoalveolar lavage fluid (BALF) of healthy controls, and patients with mild and severe COVID-19. Cells are colored by cell type identity. (**B**) UMAPs illustrating expression of *S100A12, SPP1, GAS6* and *PROS1* in BALF immune and epithelial cell populations across healthy, mild and severe COVID-19. (**C**) Bar plots illustrating normalized expression values of common mediators *S100A12, SPP1, GAS6* and *PROS1* per cell across all BALF immune and epithelial cell type. Each dot represents the average expression of these mediators per cell type per sample. Bar height is the mean value with error bars illustrating standard deviation. Healthy (n=3), mild COVID-19 (n=3) and severe COVID-19 (n=6).

Supplementary Figure 3

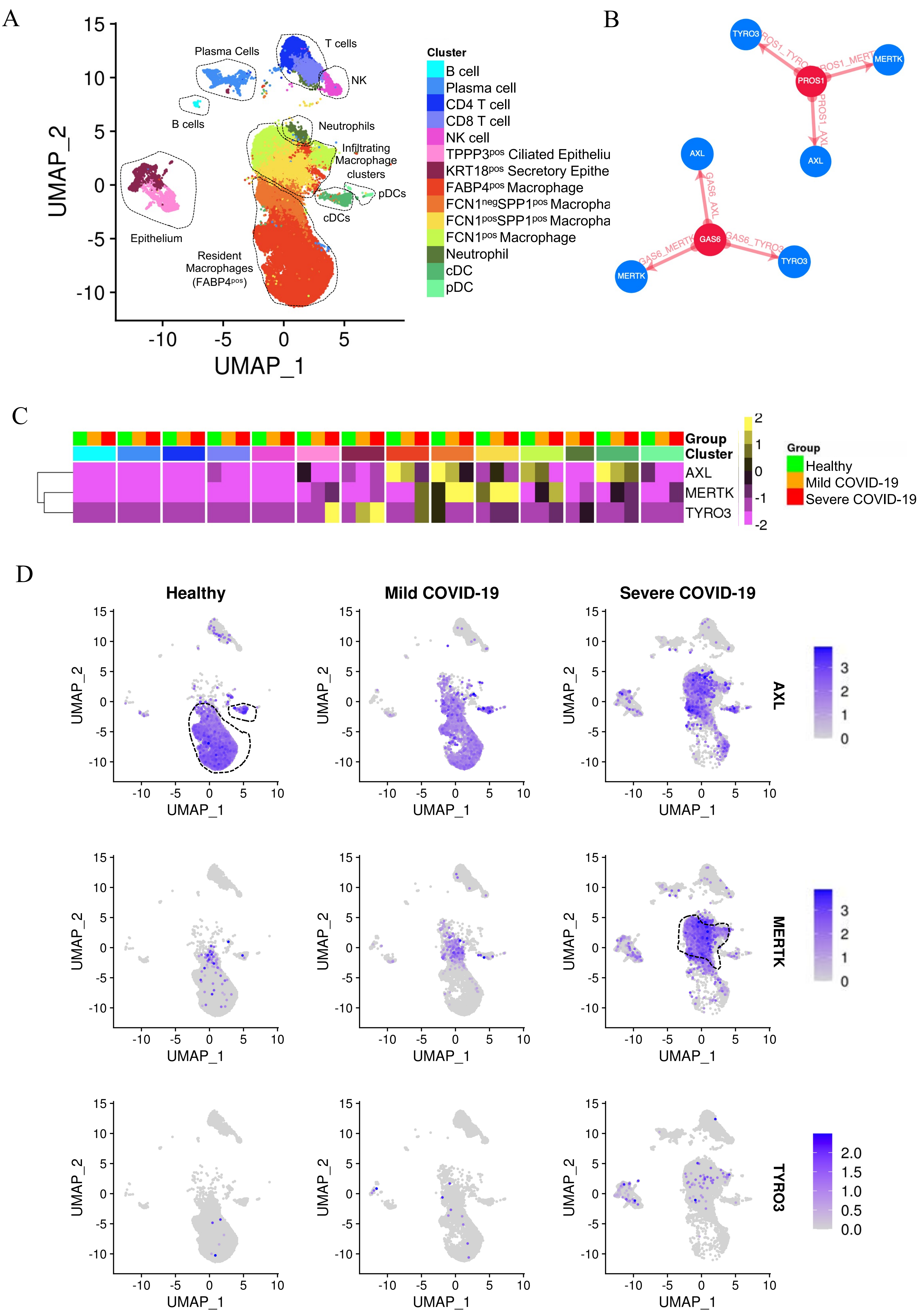

Supplementary Figure 3. Expression of identified receptors for GAS6 and PROS1 in COVID-19 BALF dataset (ref 24). (**A**) UMAP of 66,852 cells from bronchoalveolar lavage fluid (BALF) of healthy controls, and patients with mild and severe COVID19. Cells are colored by cell type identity. (**B**) Ligand-Receptor pairs for GAS6 and PROS1 identified using CellTalkDB. (**C**) Heatmap illustrating average expression by each cell type, per condition of GAS6/PROS1 shared receptors. (**D**) UMAPs illustrating expression of GAS6/PROS1 shared receptors in BALF immune and epithelial cell populations across healthy, mild and severe COVID-19.

Supplementary Figure 4

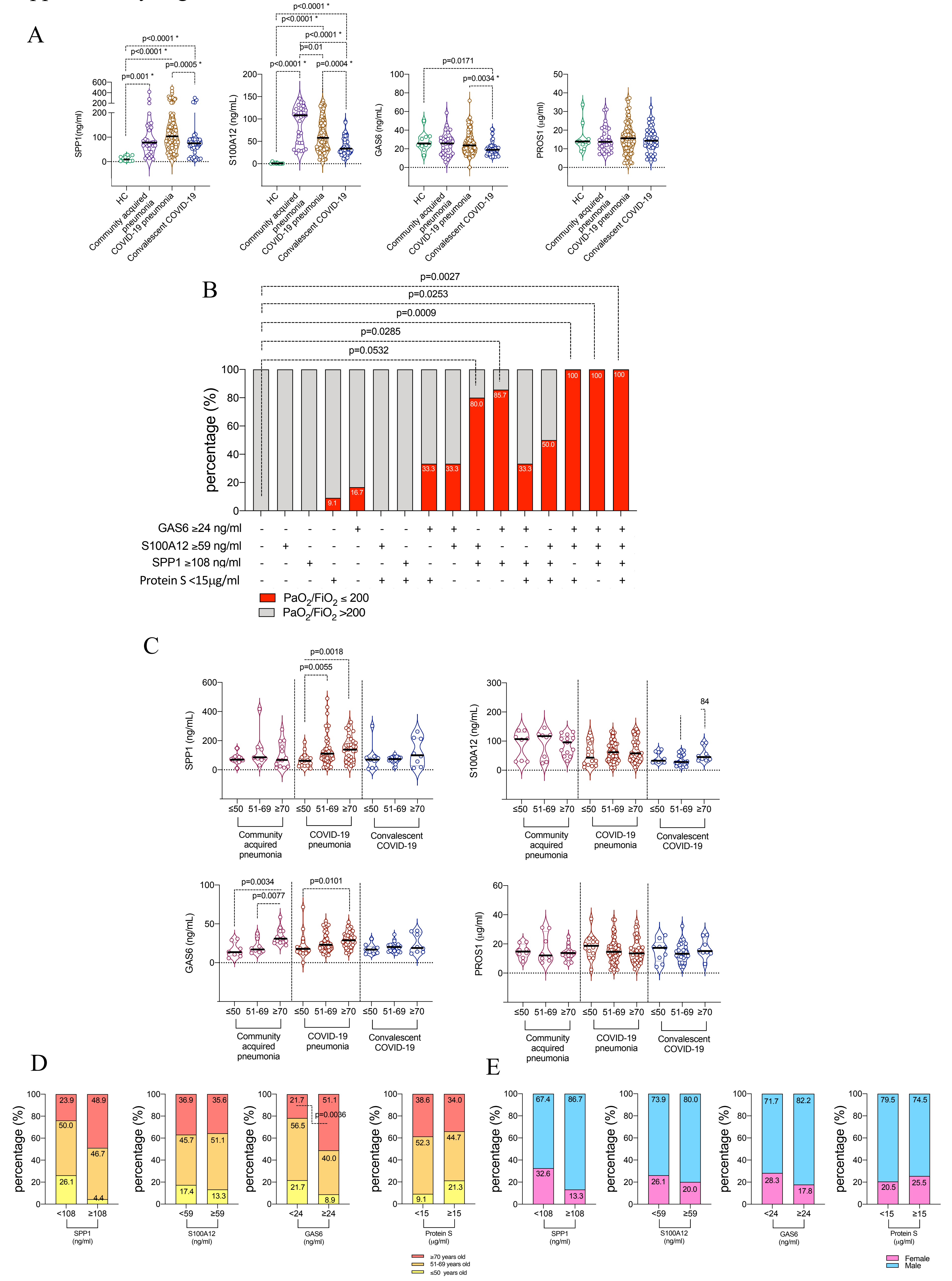

Supplementary Figure 4. Distribution of plasma levels of SPP1, S100A12, GAS6 and PROS1 in COVID-19 patients. (**A**) Levels of mediators in patients with acute COVID-19 pneumonia (n=92), patients negative for SARS-CoV-2 with community acquired pneumonia (n=29), COVID-19 convalescent patients (n=41) and healthy controls (n=10). (**B**) The combination of cut off values for SPP1, S100A12, GAS6 and PROS1 that could define the % of patients with COVID-19 pneumonia and respiratory failure ($PaO_2/FiO_2 \leq 200$). (**C**) Plasma levels of mediators in patients with acute COVID-19 pneumonia (n=92), patients negative for SARS-CoV2 with community acquired pneumonia (n=29) and COVID-19 convalescent patients (n=41) stratified based on age category. (**D**) % of patients with COVID-19 acute pneumonia (n=92) having high SPP1 (≥108 ng/ml), S100A12 (≥59 ng/ml), GAS6 (≥24 ng/ml) and PROS1 (≥15 µg/ml) plasma levels in different age categories. (**E**) % of female and male patients with COVID-19 acute pneumonia (n=92) stratified based on having high SPP1 (≥108 ng/ml), S100A12 (≥59 ng/ml), GAS6 (≥24 ng/ml) and PROS1 (≥15 µg/ml) plasma levels. *One-way ANOVA (Kruskal-Wallis test) with Dunn's correction for multiple comparisons or two-sided Mann-Whitney was used, exact p-values are provided on the graphs.

Supplementary Figure 5

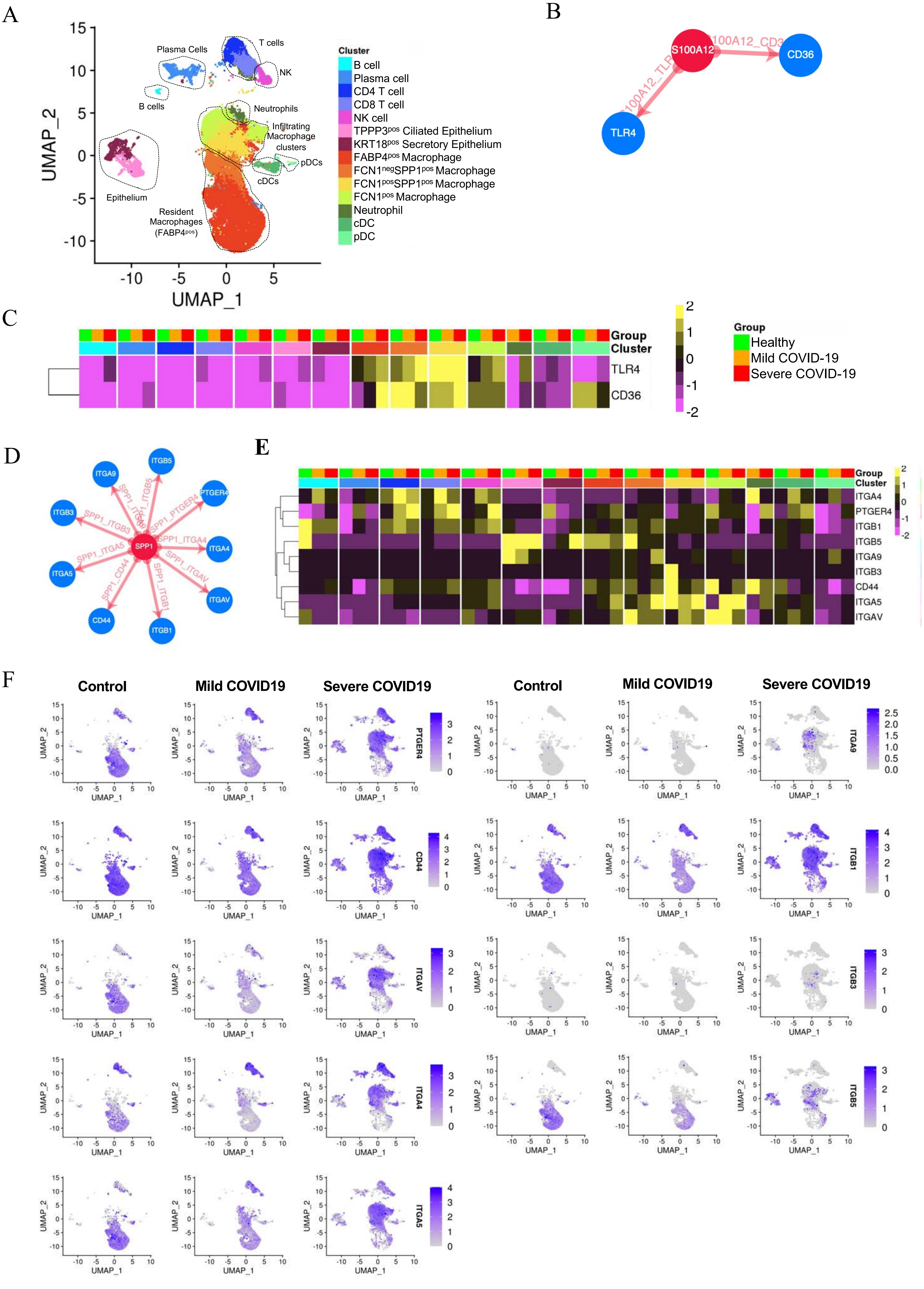

Supplementary Figure 5. Expression of identified receptors for S100A12 and SPP1 in COVID-19 BALF dataset (ref 24). (**A**) UMAP of 66,852 cells from bronchoalveolar lavage fluid (BALF) of healthy controls, and patients with mild and severe COVID-19. Cells are colored by cell type identity. (**B**) Ligand-Receptor pairs for S100A12 identified using CellTalkDB. (**C**) Heatmap illustrating average expression by each cell type, per condition of S100A12 receptors. (**D**) Ligand-Receptor pairs for SPP1 identified using CellTalkDB. (**E**) Heatmap illustrating average expression of SPP1 receptors by each cell clusters, per condition. (**F**) UMAPs illustrating expression of SPP1 receptors in BALF immune and epithelial cell populations across healthy, mild and severe COVID-19.

Supplementary Figure 6

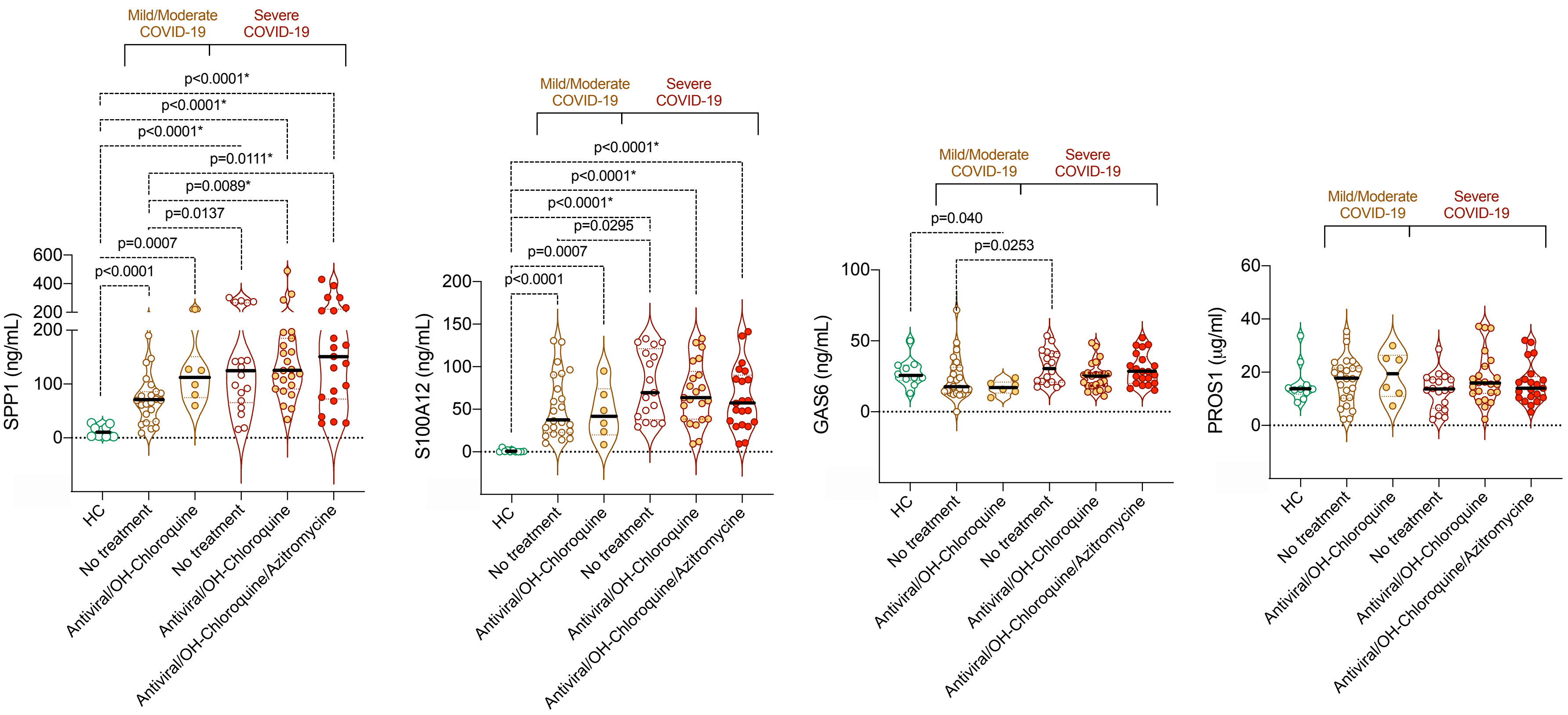

Supplementary Figure 6. Levels of SPP1, S100A12, GAS6 and PROS1 in COVID-19 patients stratified based on pharmacological treatments. SPP1, S100A12, GAS6 and PROS1 were measured in plasma of healthy (n=10) and COVID-19 patients with mild/moderate (n=29) or severe (n=63) disease. * One-way ANOVA (Kruskal-Wallis test) with Dunn's correction for multiple comparisons or two-sided Mann-Whitney was used, exact p-values are provided on the graphs.

Supplementary Figure 7

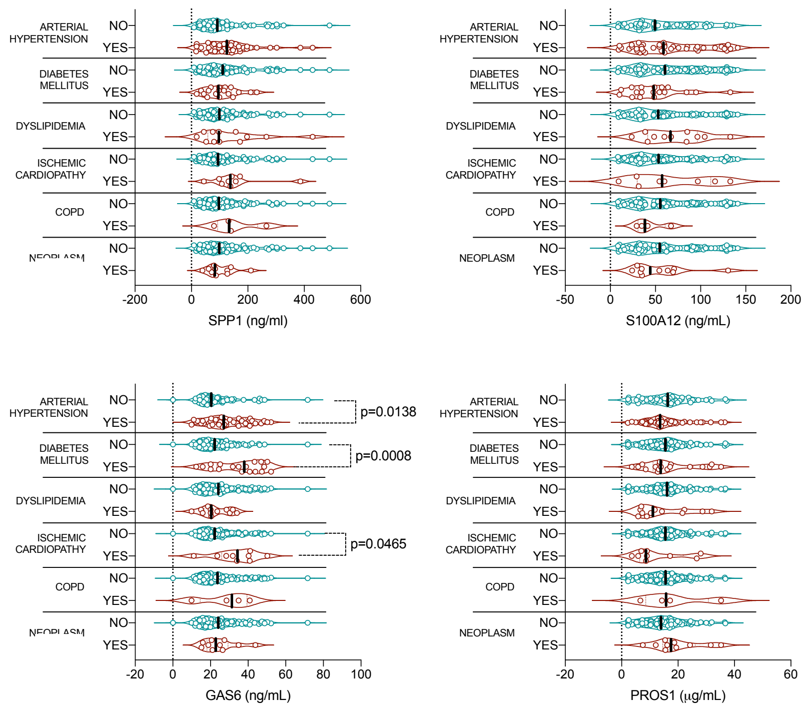

Supplementary Figure 7. Levels of SPP1, S100A12, GAS6 and PROS1 in COVID-19 patients with different comorbidities. Plasma levels of mediators in patients with acute COVID-19 pneumonia (n=92) stratified based on comorbidities (arterial hypertension, diabetes mellitus, dyslipidaemia, ischemic cardiopathy, COPD and neoplasm). Two-sided Mann-Whitney test. Exact p-values provided on the graphs.

Supplementary Figure 8

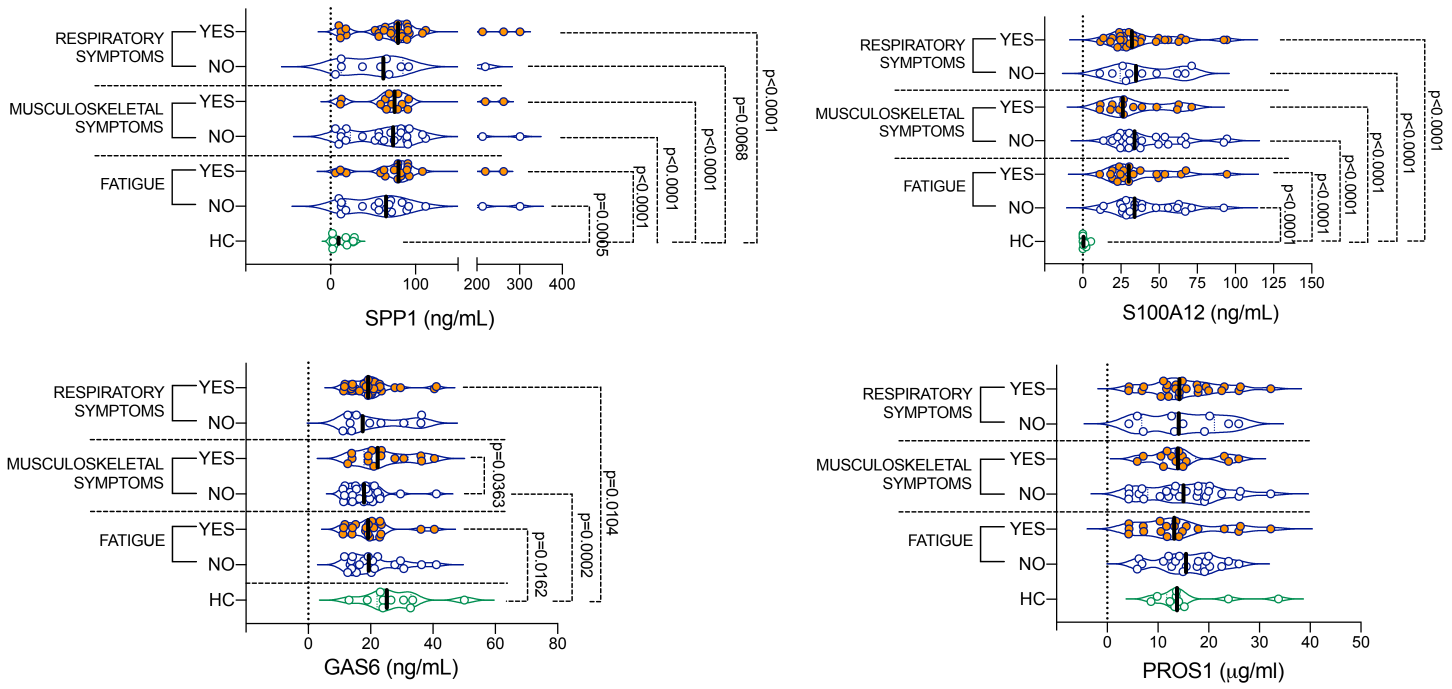

Supplementary Figure 8. Distribution of SPP1, S100A12, GAS6 and PROS1 in convalescent COVID-19 patients based on a singular post-COVID-19 symptom. SPP1, S100A12, GAS6 and PROS1 in convalescent COVID-19 patients (n=41) stratified based on experiencing a particular symptom, e.g., fatigue, musculoskeletal or respiratory symptoms persistence. Two-sided Mann-Whitney U test, exact p-values on the graphs.

Supplementary Figure 9

A) Bacterial pneumonia (SPP1/CD68/Nuclei)

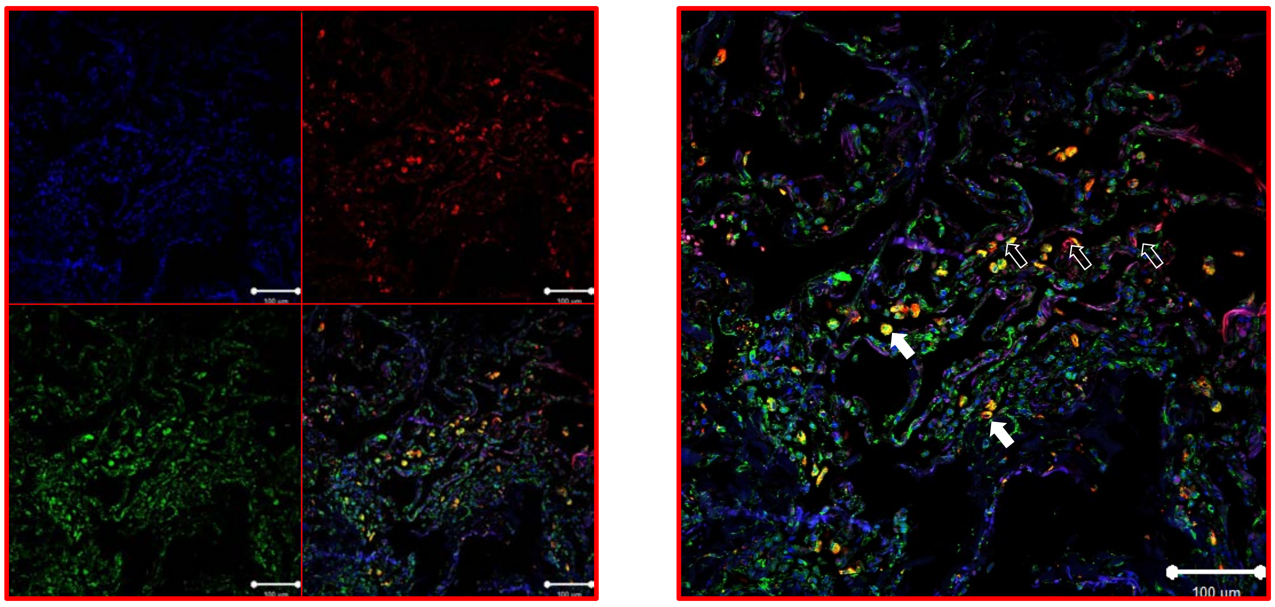

B) H1N1 pneumonia (SPP1/CD68/Nuclei)

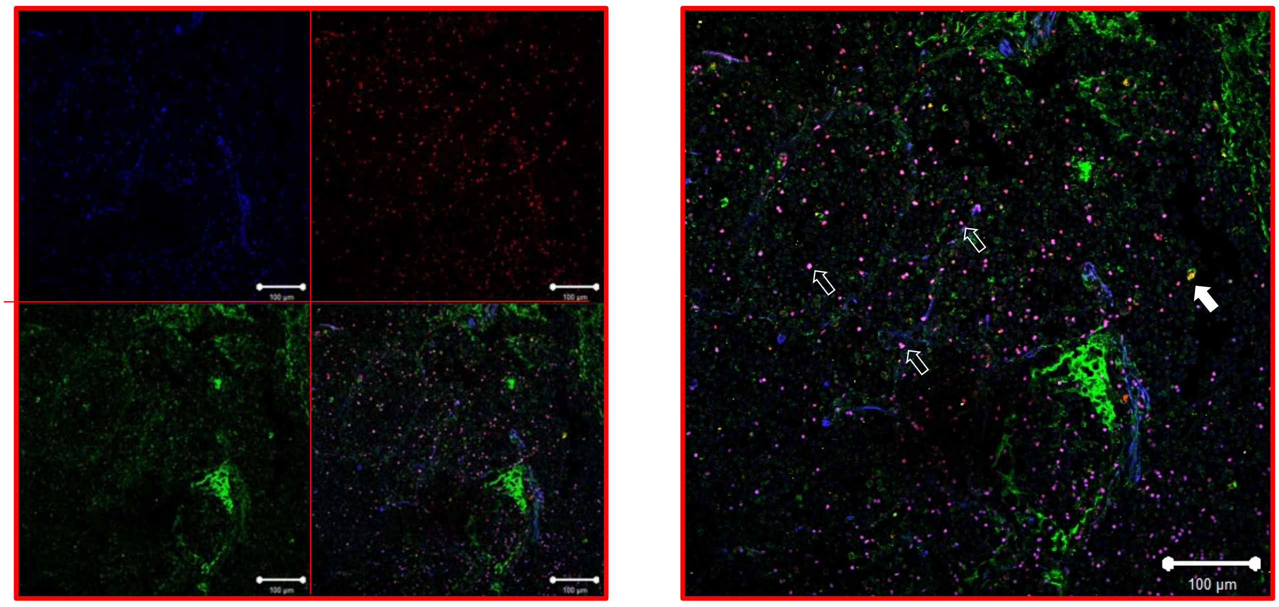

Supplementary Figure 9. SPP1 expressing macrophages are present in lungs of patients with bacteria and H1N1 induced pneumonias however to a lesser degree compared to patients with COVID-19 (Figure 5). Representative Immunofluorescence staining of lung with bacteria induced pneumonia (**A**) (n=3) and H1N1 induced pneumonia (**B**) (n=3) showing SPP1 positive and negative macrophages ($CD68^{pos}$). White solid arrows indicate double SPP1 and CD68 positive cells; white hollow arrows indicate CD68 positive and SPP1 negative cells.

Supplementary Figure 10

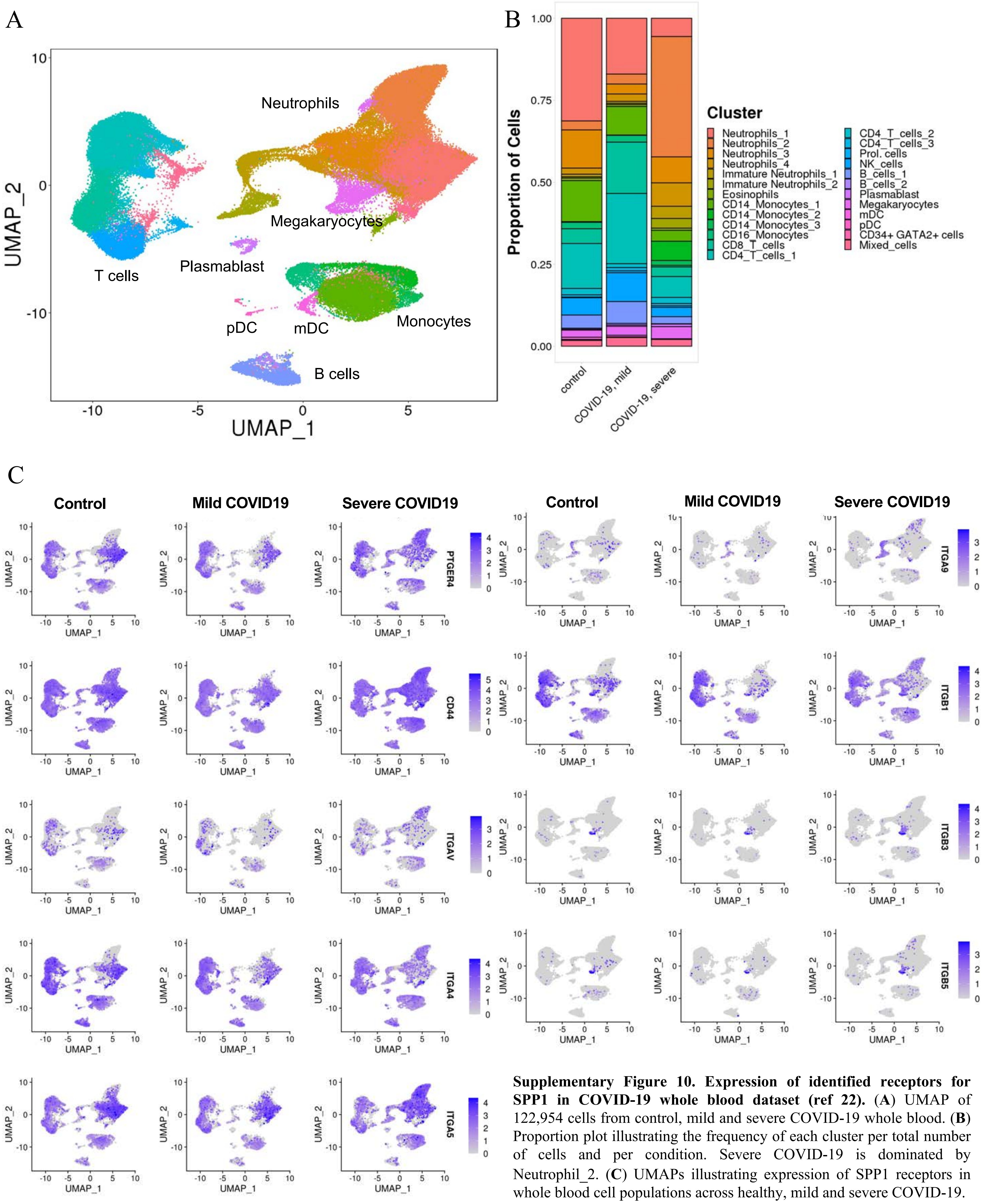

Supplementary Figure 10. Expression of identified receptors for SPP1 in COVID-19 whole blood dataset (ref 22). (**A**) UMAP of 122,954 cells from control, mild and severe COVID-19 whole blood. (**B**) Proportion plot illustrating the frequency of each cluster per total number of cells and per condition. Severe COVID-19 is dominated by Neutrophil_2. (**C**) UMAPs illustrating expression of SPP1 receptors in whole blood cell populations across healthy, mild and severe COVID-19.

Supplementary Figure 11

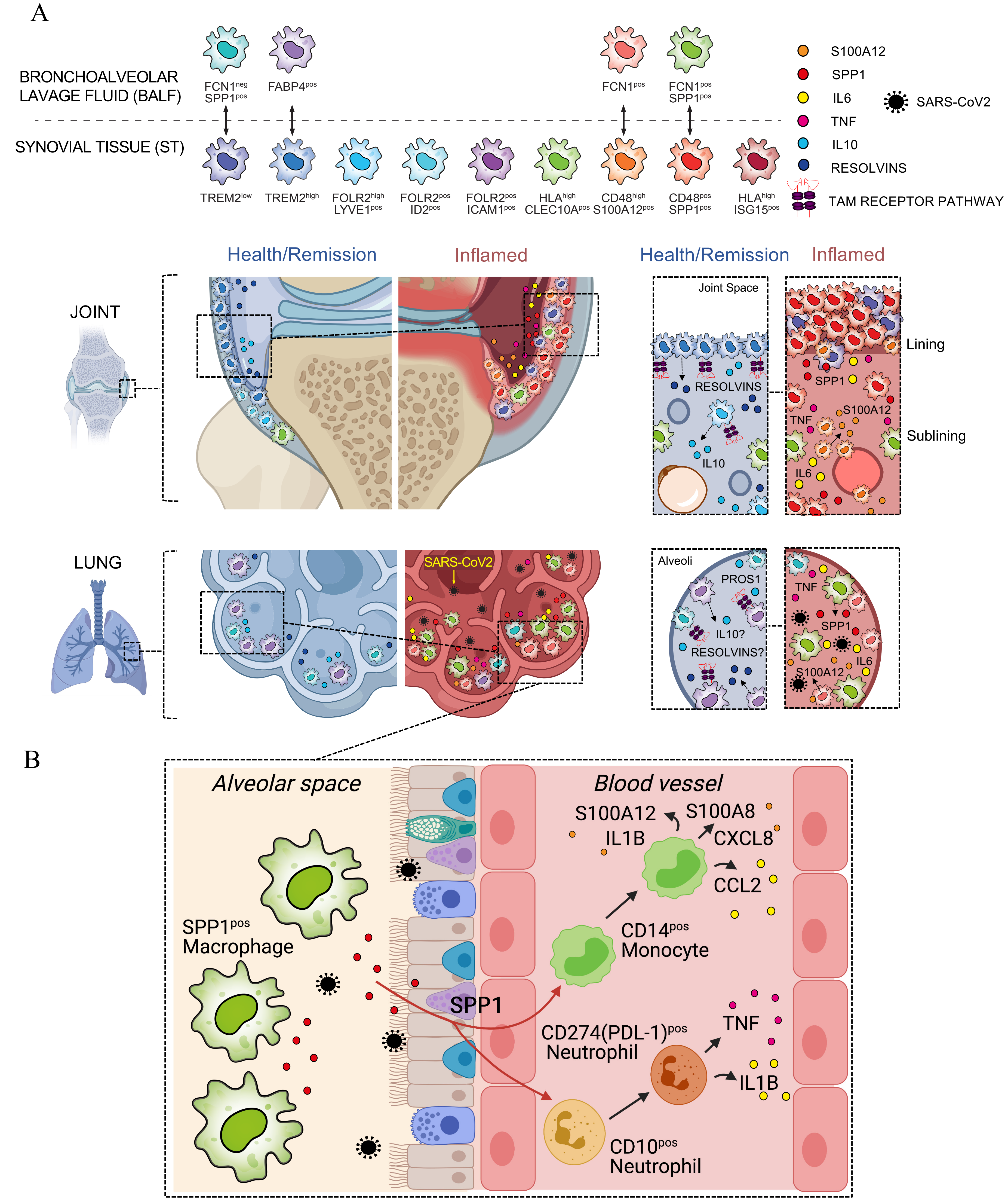

Supplementary Figure 11. COVID-19 and RA share pathogenic and resolving myeloid pathways. A) Bronchoalveolar (BALF) and synovial tissue (ST) macrophages are heterogenous. Healthy lung contains FABP4^{high} homeostatic alveolar macrophages (AM) that maintain efficient gas exchange, and a smaller FABP4^{low}SPP1^{pos} cluster with an as-yet unknown function. During SARS-CoV-2 infection, distinct FCN1^{pos} and FCN^{pos}SPP1^{pos} macrophage clusters emerge in alveoli replacing the homeostatic AM. In healthy joints, predominant TREM2^{high} and TREM2^{low} with a contribution of FOLR2^{pos}LYVE1^{pos} macrophage clusters form the synovial lining layer. The sublining layer is populated by FOLR2^{pos}LYVE1^{pos}, FOLR2^{pos}ID2^{pos}, FOLR2^{pos}ICAM1^{pos} macrophage clusters, and myeloid CLEC10a^{pos}MHCII^{high} dendritic cells. During RA, new CD48^{high}S100A12^{pos} and CD48^{pos}SPP1^{pos} ST macrophage clusters emerge, and these are the main producers of pro-inflammatory mediators and induce pathogenic changes in adjacent stromal tissue. In COVID-19, the BALF FCN1^{pos} and FCN^{pos}SPP1^{pos} macrophage clusters transcriptionally resemble these pathogenic ST CD48^{high}S100A12^{pos} and CD48^{pos}SPP1^{pos} clusters. Disease remission in RA is associated with the regulatory functions of TREM2^{high} and FOLR2^{pos}LYVE1^{pos} macrophage clusters that produce inflammation-resolving mediators (resolvins and IL-10) and induce homeostatic repair in adjacent stromal cells, mediated by MerTK. MerTK is a member of the tyrosine kinase receptor TAM family (TYRO, AXL, MerTK), and ligating TAM receptors by ligands GAS6/PROS1 forms a homeostatic brake on inflammation. Healthy alveolar FABP4^{pos} macrophages transcriptionally resemble arthritis resolving TREM2^{high} macrophages, suggesting potentially similar homeostatic mechanisms. These alveolar FABP4^{pos} macrophages constitutively express *AXL* and the MerTK ligand *PROS1*. Expression of these substantially decreases with disease severity. **B)** Pathogenic RA/COVID-19 shared FCN^{pos}SPP1^{pos} cluster localises in alveoli of COVID-19 lung and its key marker, SPP1 is significantly increased in patients with severe disease, positively correlates with respiratory distress and persist into patient' convalescence phase. SPP1 drives pro-inflammatory activation of CD14^{pos} monocytes and development of PD-L1^{pos} pathogenic neutrophils, both hallmarks of severe COVID-19. This Figure was prepared using BioRender licence of University of Glasgow.
